# Multiplex K‑Edge Nanoprobe Imaging Enables 3D Volumetric Molecular Phenotyping of Human Hematoma Membranes

**DOI:** 10.1021/acsnano.6c05415

**Published:** 2026-09-14

**Authors:** Nivetha Gunaseelan, Pranay Saha, Moumita Moitra, Shraddha Krishnakumar, Parikshit Moitra, Uttam Bodanapally, Dheeraj Gandhi, Thorsten Fleiter, Bizhan Aarabi, Dipanjan Pan

**Affiliations:** † Department of Biomedical Engineering, Nuclear Engineering, Materials Science and Engineering, 8082 The Pennsylvania State University, University Park, Pennsylvania 16802, United States; ‡ Huck Institutes of Life Sciences, The Pennsylvania State University, University Park, Pennsylvania 16802, United States; § Department of Pediatrics, Centre of Blood Oxygen Transport & Hemostasis, 486382 University of Maryland Baltimore School of Medicine, Baltimore, Maryland 21201, United States; ∥ Department of Diagnostic Radiology and Nuclear Medicine, 12264 University of Maryland Medical Center, Baltimore, Maryland 21201, United States; ⊥ Center for Metabolic Imaging and Therapeutics (CMIT), University of Maryland School of Medicine, Baltimore, Maryland 21201, United States; # Neurosurgery, University of Maryland Medical Center, Baltimore, Maryland 21201, United States

**Keywords:** K-edge nanoprobes, multiplexed molecular imaging, human tissue phenotyping, brain hematoma, machine learning, spectral photon-counting computed tomography

## Abstract

Spectral photon-counting computed tomography (SPCCT) enables material-resolved imaging through K-edge discrimination, yet its application to multiplexed molecular mapping of human surgical specimens remains underexplored. Here, we report a clinically relevant multiplex K-edge imaging strategy and demonstrate its utility directly in human chronic subdural hematoma (CSDH) membranes. SPCCT-derived human data was obtained by treating patient tissues with a rationally designed and engineered dual K-edge nanopair composed of hafnium oxide (K-edge 65.3 keV) and gold (K-edge 80.3 keV) nanoparticles to simultaneously and ratiometrically map angiogenic (α_v_β_3_) and fibrotic (fibrin) markers in the CSDH microenvironment. This nanopair provides optimized spectral separation necessary for multiplexed molecular phenotyping in human samples with high accuracy. Guided by signal separation physics, the nanopair was designed to minimize energy bin overlaps and maximize the signal-to-noise ratio in quantitative material decomposition maps. Using a calibrated phantom protocol on a Medipix3RX-based SPCCT system, we achieved accurate quantification of both elements at low millimolar concentrations. Surface-engineered hafnium nanoprobes functionalized with cyclic RGD peptides and gold nanoprobes conjugated to antifibrin antibodies enabled target-specific accumulation in distinct pathological microenvironments. Human data was analyzed as three-dimensional multicolor reconstructions that revealed simultaneous, voxel-level mapping of spatially distinct angiogenic and fibrotic microenvironments, which were validated by machine-learning-assisted immunohistochemistry quantification. These findings demonstrate the utility of a nanomaterial-driven, multiplex K-edge imaging framework for volumetric molecular phenotyping of human tissues and advance SPCCT toward quantitative, multicontrast translational imaging.

## Graphical abstract

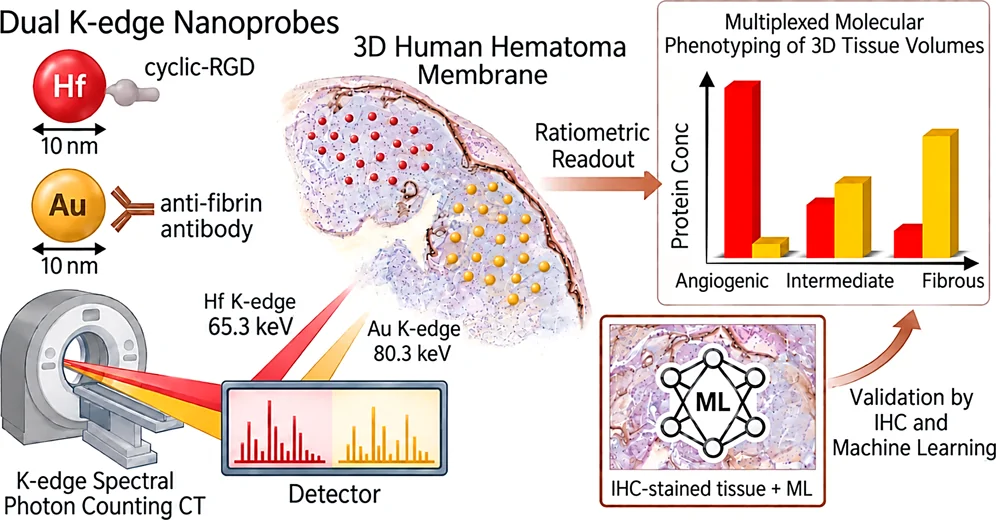

## Introduction

Three-dimensional (3D) molecular characterization of intact biological tissues remains a fundamental challenge in translational imaging.^1^ Conventional computed tomography (CT) provides high-resolution anatomical information but lacks molecular specificity,^2^ while histopathology offers molecular insight at the expense of volumetric integrity and spatial continuity.^3^ Bridging this gap requires imaging strategies capable of resolving molecular composition within intact 3D specimens in a quantitative and multiplexed manner.^4^ Spectral photon-counting computed tomography (SPCCT) offers such an opportunity by enabling energy-resolved X-ray detection and material decomposition through K-edge discrimination.^5^ Unlike conventional energy-integrating CT, photon-counting detectors bin individual photons by energy, allowing high-atomic-number (high-Z) elements to be distinguished based on abrupt increases in attenuation at their characteristic K-edge energies.^6–8^ This capability positions SPCCT as a powerful platform for multicolor molecular imaging, provided that contrast agents are rationally engineered to exploit spectral separation within the clinical X-ray spectrum.

Recent advances in nanomaterial design have expanded the repertoire of high-Z-contrast agents for K-edge imaging.^9,10^ However, multiplex molecular mapping remains constrained by spectral crosstalk, limited energy bin resolution, and suboptimal signal-to-noise ratios when multiple elements are imaged simultaneously. Rational selection of K-edge materials with sufficient spectral separation is therefore critical to minimizing overlap between adjacent energy thresholds and to preserving quantitative accuracy in material decomposition (MD) reconstructions.^11^ Engineering nanoprobe pairs with strategically positioned K-edges within the diagnostic X-ray range enables broader energy bin allocation, reduced charge-sharing artifacts, and enhanced voxel-level discrimination. When combined with molecular targeting ligands, such spectrally optimized nanopairs offer a route toward ratiometric, multiplexed phenotyping of complex tissue microenvironments.^12^

Chronic subdural hematoma (CSDH) presents a clinically relevant and biologically heterogeneous model system to evaluate such multiplex imaging strategies. CSDH is a common neurosurgical condition characterized by progressive membrane formation driven by intertwined angiogenic and fibrotic processes.^13,14^ Early-stage membranes are dominated by fragile, α_v_β_3_-integrin-expressing neovasculature with high permeability, whereas later-stage lesions exhibit increased fibrin deposition, clot organization, and reduced vascular remodeling.^15–18^ These pathological transitions influence recurrence risk following surgical evacuation, yet current imaging modalities primarily assess morphology or contrast leakage without resolving the underlying molecular microenvironment.^19–21^ Utilizing human samples to develop a volumetric method capable of simultaneously mapping angiogenic and fibrotic domains within intact excised membranes could provide mechanistic insight into disease stage and tissue remodeling.^12,22^

Here, we report a physics-informed dual K-edge nanoprobe strategy for multiplex molecular phenotyping of CSDH membranes using SPCCT-derived human data. We rationally selected hafnium and gold as a spectrally separated high-Z nanopair positioned within the diagnostic X-ray spectrum to minimize bin overlap and optimize signal discrimination. Hafnium oxide nanoprobes were synthesized via a sol–gel microemulsion route and surface-functionalized with cyclic RGD (cRGD) peptides targeting α_v_β_3_ integrin, while gold nanoprobes were conjugated with antifibrin antibodies to label organized clot components. Using a Medipix3RX-based SPCCT platform operating in charge-sharing mode, we developed a calibrated acquisition protocol enabling simultaneous detection and quantification of both elements at low concentrations. Three-dimensional MD maps of patient-derived tissue blocks revealed spatially distinct angiogenic and fibrotic microdomains, which were validated using machine-learning (ML)-assisted immunohistochemical quantification.

We have previously demonstrated that K-edge-based SPCCT imaging can be used to do a comparative analysis of the expression profiles of two biomarkers to effectively stage disease in patient samples.^23–26^ Although a variety of high atomic number elements can be considered as contrast agents for SPCCT imaging, a proper selection of materials according to their K-edge energies improves material discrimination and provides high accuracy at low binding concentrations of the contrast agents at low protein expression levels. The selection of two materials with larger K-edge energy separation within the CT X-ray spectrum enables the design of nanoprobes with wider energy bins to optimize the signal-to-noise ratio (SNR) in the K-edge ratio parametric maps. This helps in the precise identification of multiple contrast agents in biological tissues when simultaneously seeking different targets. Therefore, in this study, we have demonstrated that the unique pairing of hafnium (Hf; K-edge = 65.3 keV, Z = 72) and gold (K-edge = 80.3 keV, Z = 79) as the dual K-edge nanopair provides an ideal K-edge separation minimizing the overlap between the two SPCCT energy bins, thus enabling seamless multiplexing for tissue color mapping (Scheme 1). Tissues from diseased patients were carefully treated with this nanopair targeted to two different CSDH-specific targets to better understand their pathology at the molecular level. These tissues were also preanalyzed using dual-energy (DECT) quantification of iodine leak through the immature capillaries in the external membrane for prognosis at clinical presentation. The SPCCT analysis of the tissues was further corroborated with tissue histopathology to validate the protein expression levels. Additionally, the quantification of protein expression patterns in the histological data was conducted by using a proprietary ML method to enhance the validation of the SPCCT data.

**Scheme 1:**
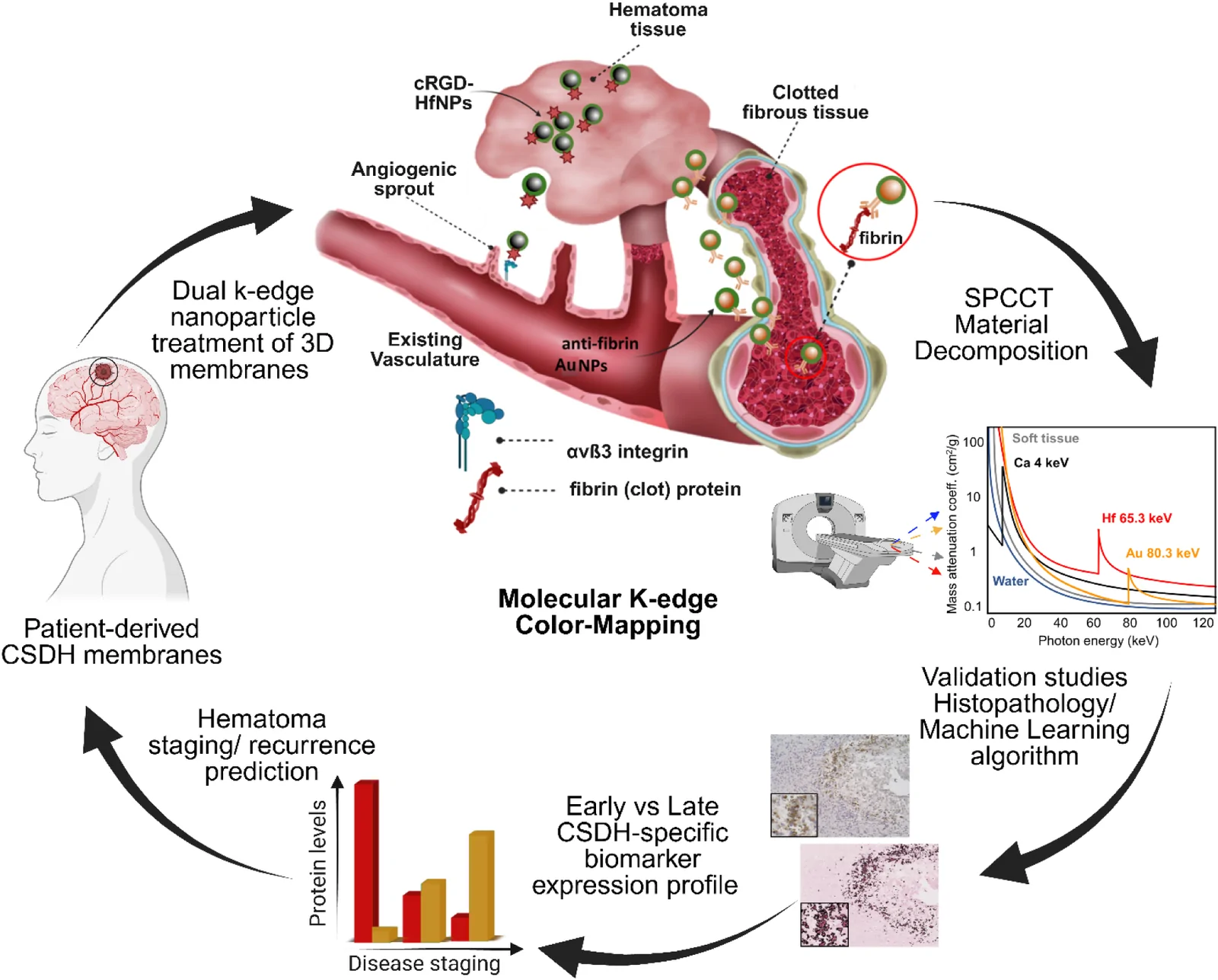
Process Flow for K-Edge Color Mapping and Molecular Analysis of Human Chronic Subdural Hematoma Tissues Using Dual Nanoprobes Based on Hafnium and Gold Targeted to Two Clinically Relevant CSDH Markers of Interest (Created in BioRender)[Fn sch1-fn1]

i The SPCCT results are validated using gold standard immunohistochemical tissue analysis and ML algorithm-based quantification techniques.

## Results and Discussion

### Synthesis and Characterization of Surface-Modified Dual K-Edge Nanoprobes

Hafnium nanoparticles were synthesized using a previously reported base-catalyzed sol–gel water-in-oil microemulsion method with a polyethylenimine silane grafting approach.^26^ The protocol yielded tiny spherical hafnium oxide nanoparticles (HfNPs) which were surface-functionalized with cRGD peptide targeted to α_v_ß_3_ integrin to target angiogenic vessel growth in the hematoma (Figure 1a). This functionalization was carried out by first completing a ligand exchange process with succinic anhydride and then using a 1-ethyl-3-(3-(dimethylamino)­propyl) carbodiimide (EDC) and *N*-hydroxysuccinimide (NHS) coupling method which yielded c-HfNPs. Similarly, the second K-edge probe based on gold was synthesized using a previously reported modified Turkevich method.^27–29^ These gold nanoparticles (AuNPs) were surface-functionalized with antifibrin antibody targeted to the fibrin marker expressed in the fibrous clotted tissue in the hematoma. Antibody functionalization was achieved using DTSP (3,3′-dithiobis­(succinimidyl propionate), which is a thiol/disulfide-to-amine cross-linker commonly used to couple antibodies to a gold nanoparticle surface.^27^ The process led to the introduction of NHS esters on the DTSP-activated AuNP surface which enabled covalent amide linkage with the antifibrin antibody amines (EDC-NHS chemistry) for successful surface modification of the nanoparticle to produce f-AuNPs (Figure 1a). Material characterization of both nanoparticles was done using several techniques. Transmission electron microscopy (TEM) imaging was done to visualize the formation of well-dispersed spherical particles and measure the average anhydrous diameter of the synthesized hafnium and gold nanoparticles which were calculated to be 10 ± 5 nm and 10 ± 3 nm, respectively, with surface capping agents (Figure 1b,c). The anhydrous particle sizes visualized in TEM images were analyzed and represented as frequency distribution histograms in Figure 1d,e, with their respective insets showing the number-averaged hydrodynamic diameter of the resulting HfNPs (26 ± 3 nm) and AuNPs (11 ± 2 nm) determined from dynamic light scattering (DLS) measurements. Fourier transform infrared spectroscopy (FTIR) analysis was conducted to verify the successful conjugation of cRGD to HfO_2_ nanoparticles by the emergence of characteristic peptide amide features in the spectrum of HfO_2_–cRGD relative to bare HfO_2_ and free cRGD. The conjugated nanoparticles exhibit a broad band at ∼3230 cm^–1^ attributed to the amide A (N–H stretching) vibration and a pronounced band at ∼1640 cm^–1^ corresponding to the amide I (C=O stretching) mode of the peptide backbone, both absent in bare HfO_2_ and consistent with peptide amide signatures. In contrast, the amide II band observed for free cRGD near ∼1530 cm^–1^ is strongly attenuated reflecting partial masking of peptide backbone vibrations in the nanoparticle-bound state, a behavior commonly reported in surface-sensitive FTIR analyses.^30^ The presence of amide A and amide I bands supports successful immobilization of cRGD on the HfO_2_ surface (Figure 1f).

**Figure 1:**
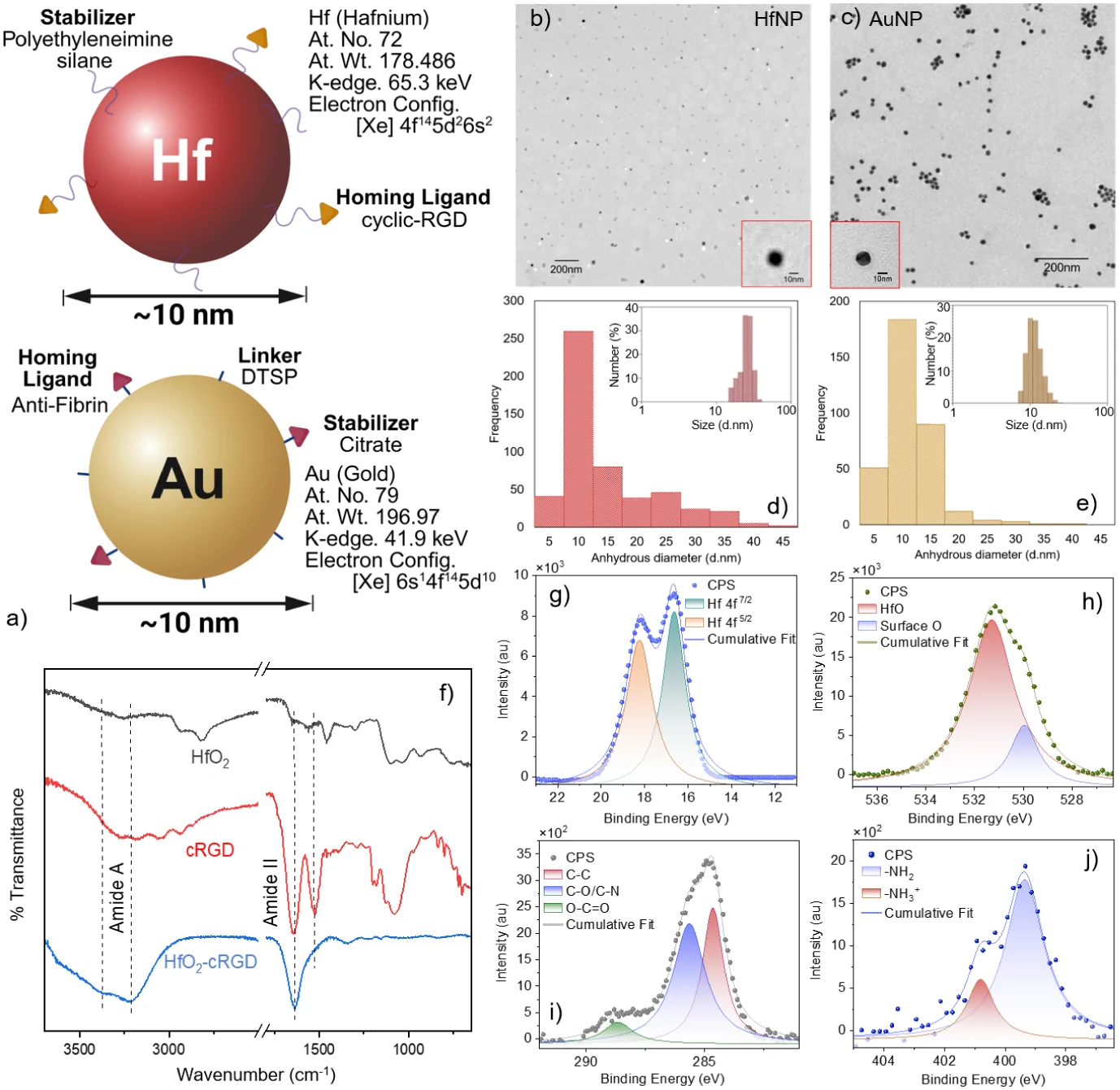
(a) Schematic representation of the spherical K-edge nanoparticles functionalized with homing ligands via EDC-mediated coupling (created in BioRender); Characterization of the synthesized nanoprobes: TEM images showing anhydrous diameters of b) HfNPs and c) AuNPs; scale bar = 200 nm; insets show a zoomed-in view of a single nanoparticle; scale bar = 10 nm; Frequency distribution histograms showing anhydrous nanoparticle diameter obtained from TEM for d) HfNPs and e) HfNPs. Both histograms demonstrate a predominance of nanoparticles with diameters around ∼10 nm with insets showing DLS measurements of HfNPs and AuNPs, respectively. These histograms show the hydrodynamic size distribution of the particles; f) attenuated total reflectance FTIR spectra of nontargeted HfO_2_ and targeted c-HfO_2_ nanoparticles compared to only cRGD spectra in the CH and fingerprint region showing successful ligand functionalization; XPS spectra of HfO_2_nanoparticles showing (g) Hf 4f, (h) O 1s, (i) C 1s, and (j) N 1s orbitals.

Furthermore, XPS analysis confirmed the characteristics of the HfO_2_ nanoparticles. The Hf 4f spectrum shows characteristic doublets at ∼16.8 eV (Hf 4f_7_/_2_) and ∼18.4 eV (Hf 4f_5_/_2_), consistent with the Hf^4+^ oxidation state and excluding metallic hafnium (Figure 1g). The O 1s spectrum was deconvoluted into a lattice oxygen (Hf–O–Hf) peak at ∼530.0 eV and a dominant higher-binding-energy surface oxygen component at ∼531.5 eV, attributed to hydroxylated and polymer-interacting (PEI) surface sites (Figure 1h). Successful PEI functionalization is evidenced by the N 1s spectrum, which displays contributions from neutral amines (−NH_2_, ∼399.4 eV) and protonated amines (−H_3_^+^, ∼401 eV), indicating partial protonation and strong interfacial interactions with HfO_2_ (Figure 1j). The C 1s spectrum further supports PEI coating, showing C–C/C–H (284.5 eV), C–N (∼286.6 eV), and minor O–C=O (∼288.5 eV) components (Figure 1i).

The percent ligand functionalization on the nanoparticle surface was quantified by both fluorescence and flow cytometry analysis to achieve maximum binding efficacy of the probes. Successful conjugation of antibodies onto HfNPs and AuNPs was first evaluated by fluorescence-based FITC- and Cy5-antibody labeling kits. To achieve this, first, the surface-modified HfNPs (c-HfNPs) and AuNPs (f-AuNPs) were purified through centrifugation and labeled with commercially available FITC and Cy5 labeling kits, respectively (Figure 2a,b). Postlabeling, they were purified using separation columns as shown. Standard curves for FITC and Cy5 were obtained to determine the percent of each ligand conjugated to the respective nanoparticles (Figures 2c,d and S1). Combining the surface conjugation data with nanoparticle number calculations yielded an estimated ligand density of ∼3 ligand molecules per HfNP which corresponds to an estimated 2% surface coverage. For AuNPs, the ligand conjugation was intentionally maintained at a nominal 1:1 nanoparticle-to-ligand ratio to account for the larger size of the antibody. These relatively low ligand densities were strategically selected to promote efficient targeting while minimizing steric hindrance and nonspecific binding within the tissue samples. Furthermore, to assess whether the ligand-conjugated nanoparticles retained their fluorescence signal following cellular exposure, flow cytometry was performed after incubation with cells.^28^ Mouse brain endothelial (bEnd.3) cells treated with fluorescent ligand-kit treated c-HfNPs and f-AuNPs displayed a pronounced rightward shift in fluorescence intensity in both FITC and Cy5 channels confirming successful cellular association of the fluorescent-tagged ligand-labeled nanoparticles (Figure 2e,f). Quantitatively, these samples showed significantly elevated mean fluorescence intensity (MFI) and increased percentages of fluorescently positive cells, consistent with efficient nanoparticle delivery mediated by ligand conjugation. In contrast, control nanoparticles processed with the same fluorescent ligand-labeling kits but without ligand conjugation (NP-only + labeling kit) exhibited minimal fluorescence both prior to and following cellular treatment (Figure S2). This observation confirms that nonspecific adsorption of fluorescent dyes to the nanoparticle surface was negligible and that stable fluorophore attachment occurred predominantly through ligand conjugation rather than direct dye–nanoparticle interactions. For ruling out NP-induced autofluorescence or background signal contributions, additional control experiments were performed using cells treated with unlabeled, ligand-free HfNPs or AuNPs which showed fluorescence profiles comparable to untreated cells. The observed fluorescence in c-HfNP- and f-AuNP-treated cells originates from ligand-bound fluorophores rather than free dye, nonspecific labeling, or nanoparticle-associated artifacts.

**Figure 2:**
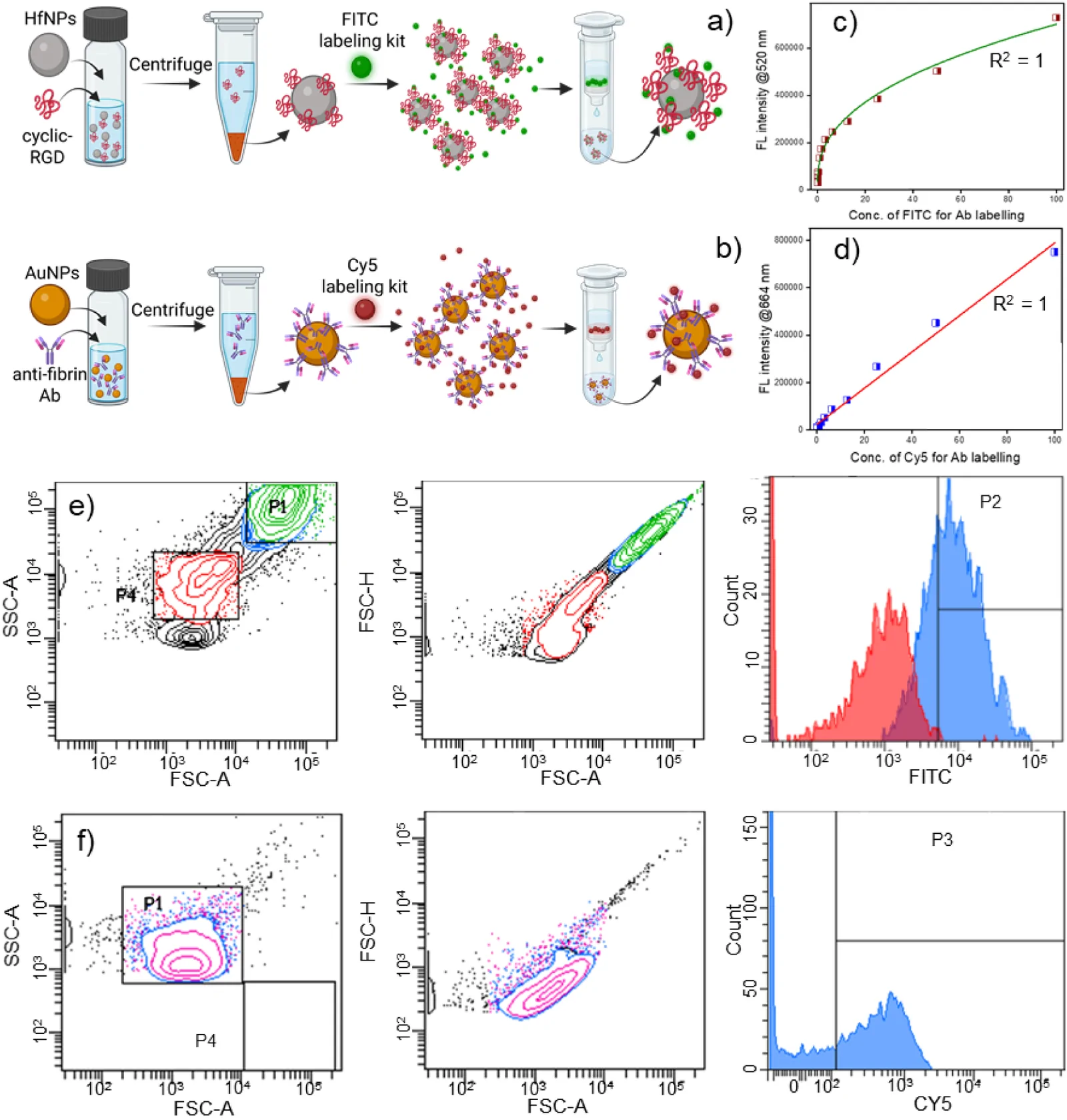
Schematic showing a) FITC labeling of c-HfNPs and b) Cy5 labeling of f-AuNPs for percent ligand functionalization analysis and optimization (created in BioRender); fluorescence standard curves for c) FITC-labeled c-HfNPs and d) Cy5-labeled f-AuNPs; flow cytometry experiments exhibited (e) 68% FITC-positive cells treated with c-HfNPs with an increase of 38% compared to as-synthesized HfNPs without ligand conjugation and f) 44% Cy5-positive cells treated with f-AuNPs with a 100% increase compared to as-synthesized AuNPs without ligand conjugation.

### Standardization of SPCCT Protocol for Multiplexed Detection of Hf and Au Nanoprobes

Currently, the morphology of CSDH membranes (Figure 3a) and their permeability are visualized using Dual Energy CT (DECT) by observing iodine contrast leaks through immature capillaries in the CSDH membranes.^31,32^ Therefore, human data was collected prior to patient surgical sample collection using DECT/DynaCT as shown in Figure 3b,c as a clinical reference. However, this visualization technique does not provide any molecular (functional) information about physiological processes such as the extent of angiogenesis or the amount of fibrous tissue (fibrinolysis) formation to gain a deeper understanding of the stage of the hematoma and therefore predict the probability of recurrence postsurgery.

**Figure 3:**
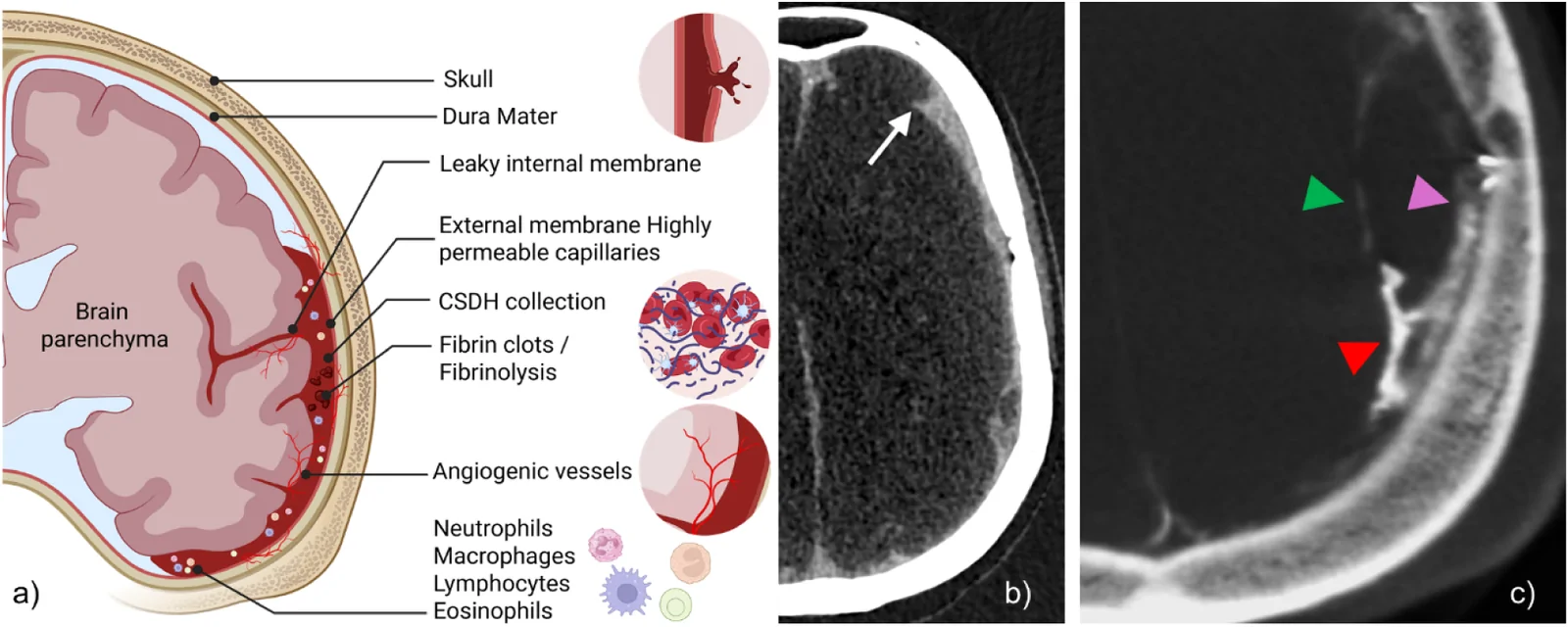
(a) Schematic representation of CSDH membranes with contributing factors for the development and sustenance of the condition, showing angiogenic new vessel growth and fibrinolysis (clot formation) processes (created in BioRender); Human data: b) Head CT image of a 71-year-old man with left CSDH. Postcontrast 5 min delayed grayscale iodine map that shows a well-defined external membrane (white arrow); c) DynaCT obtained during endovascular embolization showing the enhancement of the external membrane (pink arrow), internal membrane (green arrow), and iodine contrast leak from the membranes (red arrow).

Multiplexed SPCCT imaging with dual K-edge contrast provides an ideal platform to visualize these underlying processes, and to achieve this, an optimized imaging protocol designed to identify and delineate both agents simultaneously with maximum accuracy is required. To achieve this, a phantom study was designed for simultaneous detection of Hf and Au materials using the MARS Microlab 5 × 120 small-bore SPCCT scanner (MARS Bioimaging Ltd., New Zealand). The scanner is equipped with advanced cadmium–zinc–telluride (CZT)-assembled Medipix3RX detector that is both energy resolving and photon-counting providing MD capabilities and true quantitative images. The protocol was developed to retain high levels of soft tissue contrast while accurately identifying low concentrations of Hf and Au simultaneously by taking advantage of their well-separated K-edge energies to avoid signal overlap while maximizing signal intensity. The protocol was designed specifically to achieve the best soft tissue contrast while also maximizing counts to deliver the best MD calibration. The phantom is composed of a PMMA (poly­(methyl methacrylate)) MARS material phantom loaded with 2 mL Eppendorf tubes containing varying concentrations of Hf (1, 2, 4, and 8 mg mL^–1^), Au (1, 2, 4, and 8 mg mL^–1^), calcium (35, 70, and 140 mg mL^–1^), water, and lipid as shown in Figure 4a. The 1–8 mg mL^–1^ calibration range was deliberately selected to cover low-end concentrations capturing the detection limit of the SPCCT system and high-end concentrations to assess saturation and preserve linearity, thus acting as a dynamic range of the MD algorithm.^7,33^ Since targeted binding is spatially heterogeneous due to nonuniform density of the target antigens, this produces hotspots of nanoparticle accumulation. Thus, the calibration range helps capture this expected voxel-averaged signal output due to localized targeted particle accumulation in the CSDH tissues. The protocol’s acquisition parameters were carefully optimized and set to a tube voltage of 118 kVp, a tube current of 61 μA, and an exposure time of 160 ms. Table S1 shows all the optimized parameters used in developing the imaging protocol. The energy bin thresholds were deliberately set to 7–35, 35–45, 45–55, 55–65, and 65–118 keV to account for the K-edge energies of both Hf and Au in Charge Summing Mode (CSM) as shown in Figure 4e. The energy binning structure was chosen in order to balance the detection of the K-edge and the linear attenuation curve of each material that allows for the best separation of the three materials.

**Figure 4:**
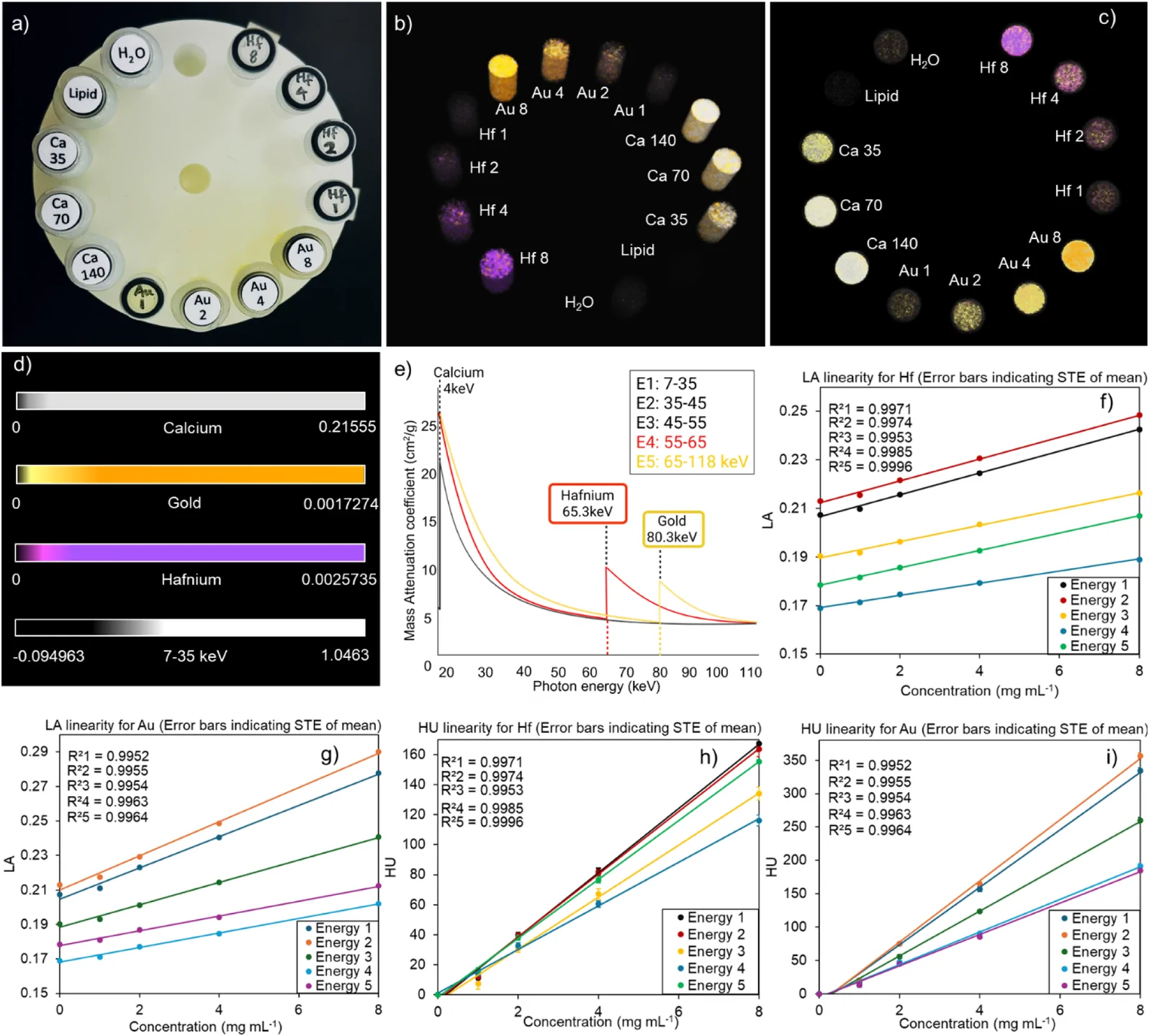
(a) MARS material phantom loaded with varying concentrations of hafnium and gold with calcium, lipid, and water for protocol development/optimization and material decomposition in biological samples. The concentration ranges used for both hafnium and gold were 1, 2, 4, and 8 mg mL^–1^; b) 3D reconstructed view of the phantom. Materials have been identified by the MIQ algorithm and color-coded using the MARS Vision software, hafnium (purple), gold (yellow), and calcium (white), to show the accuracy in material decomposition and exact location of the materials in the phantom; c) 2D axial view of the phantom scan; d) color map showing MIQ algorithm-based identification of the materials and their concentrations; e) graph showing K-edge separation between the Hf and Au metal pair (created in BioRender). This shows how Ca, Hf, and Au are distributed across the SPCCT energy bins providing minimal signal overlap and high accuracy for simultaneous detection; phantom analysis showing linearity of attenuation of HfNP in each energy bin. f) Linear attenuation. h) Hounsfield unit. Phantom analysis showing linearity of attenuation of AuNPs in each energy bin. g) Linear attenuation. (i) Hounsfield unit. The regression curves show that the SPCCT response is linear for both materials demonstrating accuracy in material identification. Data is presented as mean ± standard deviation (*N* = 5).

Prior to scanning, pixel masking was applied to remove noisy pixels, and postscanning, the MARS Algebraic Reconstruction Technique (mART) was used to reconstruct the phantom scan data.^34^ The image protocol parameters were carefully optimized to achieve the highest possible accuracy in detecting three materials simultaneously with the MARS material identification and quantification (MIQ) algorithm achieving minimum accuracy levels of ∼84% for Hf, ∼81% for Au, and ∼87% for Ca with minimal crosstalk between materials. This indicates a high enough confidence to detect very low concentrations simultaneously within biological tissue samples. The limit of detection (LOD) was experimentally determined by analyzing the performance accuracy of the system at a 1–8 mg mL^–1^ concentration range with Hf at 1 mg mL^–1^ and Au at 4 mg mL^–1^ using the optimized imaging protocol. Hf LOD was observed to be near the lower bound of detectability with improved performance observed at ≥4 mg mL^–1^ suggesting more robust spectral separation at higher concentrations. Table S2 shows the percent quantification achieved across the material phantom vials using the optimized protocol. However, future efforts are required to increase the accuracy levels that can be achieved using SPCCT systems for 3-material decomposition. The reconstructed 3D and axial images of the final phantom loaded with Hf and Au in the calibrated concentration ranges with Ca (clot calcification), water and lipid to mimic the tissue samples are shown in Figure 4b and c, respectively. The color map (Figure 4d) represents the MIQ algorithm-based MD used to delineate each material in the phantom accurately. To analyze the phantom data, linear attenuation (LA) and standard deviation for each concentration and material in the phantoms were quantified and converted to Hounsfield Units (HU) by manually selecting a region of interest (ROI) over each vial. Linear regression analysis was used to determine the relationship between HU and material concentration to obtain a linear response curve. Several axial slices were selected from each energy bin, and the relationship between HU and the energy bin was evaluated graphically, and the spectral response was recorded. The LA and HU linearity curves show high R^2^ values for Hf, Au, and Ca, which indicate that the protocol has been optimized to achieve a relatively high accuracy for 3-material decomposition (Figures 4f–i and S3). This was achieved by careful selection of the K-edge nanopair providing decent spectral separation across the energy bins. However, the study is limited by the vendor-defined system MD threshold, and some minimal cross-signal is observed in the low-concentration vials. Future work will therefore focus on improving the accuracy of multimaterial detection in this low-concentration regime. However, such optimization falls beyond the scope of the present nanotechnology-focused study.

### Design of In Vitro 3D Spheroid Models and Ex Vivo Clot Model Mimicking a Hematoma Membrane Environment for Evaluating the Binding Efficacy of Hf and Au Nanoprobes

To evaluate the binding efficacy of the nanoparticles to the markers of interest, we developed an *in vitro* model expressing angiogenic markers and an *ex vivo* clot model to mimic the fibrous hematoma membrane microenvironment. For the *in vitro* model, bEnd.3 cell line was used to grow 3D spheroids using Nunclon Sphera well plates since the architecture better recapitulates the hematoma membrane-like microenvironment than a 2D culture, enabling assessment of α_v_β_3_-directed nanoparticle penetration and binding under conditions relevant to the hematoma membrane milieu (Figure 5a). bEnd.3 is a highly relevant cell line since it naturally expresses high levels of the α_v_ß_3_ integrin, and the 3D spheroids also help mimic gradients (oxygen/nutrients) and ECM/cell–cell contacts that affect integrin presentation and nanoparticle penetration.^35^ The bEnd.3 *in vitro* study is aimed at illustrating the varying intake of c-HfNPs (targeted) and bare HfNPs (nontargeted) resulting from alterations to the nanoparticle surface. Cells treated with equimolar quantities of both particles exhibited significantly higher uptake of c-HfNPs compared to the nontargeted bare HfNPs, indicating the nanoprobe’s strong targeting efficacy with minimal nonspecific binding (Figure 5b–d). The treated cell pellets were imaged using MARS SPCCT, and the differential uptake between treatment groups was visualized.

**Figure 5:**
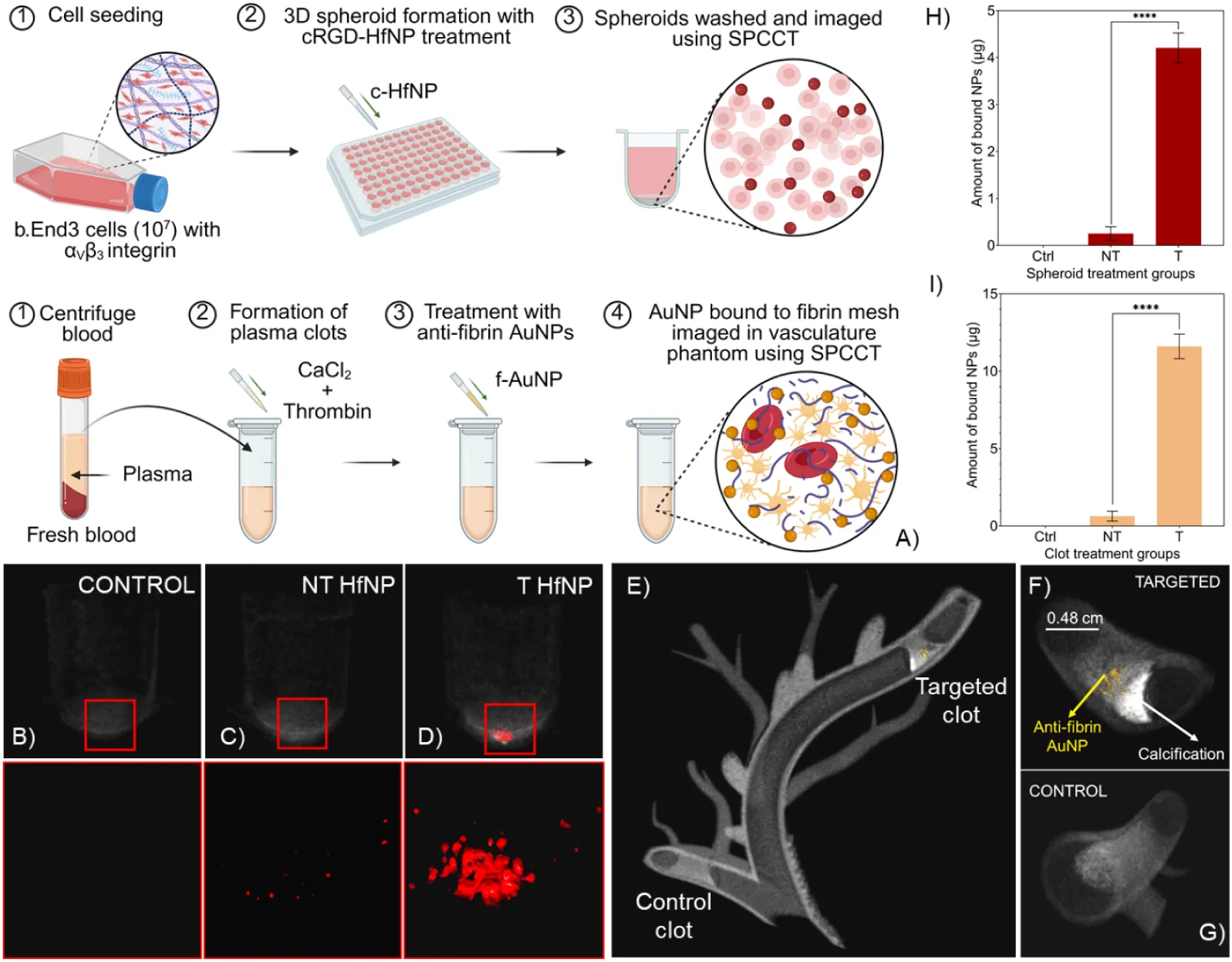
Nanoprobe binding efficacy analysis. A) Schematics showing *in vitro* bEnd.3 spheroid cell culture with α_v_ß_3_ integrin expression and treatment with a c-HfNP nanoprobe for CSDH angiogenesis modeling and *ex vivo* plasma clot formation and treatment with an f-AuNP nanoprobe for CSDH fibrous clot tissue modeling (created in BioRender); MD-applied SPCCT 3D rendering of a B) control bEnd.3 spheroid cell pellet with no NP treatment, C) treatment with nontargeted HfNPs, and D) treatment with targeted c-HfNPs; zoomed-in images (below) showing signals obtained from selective binding of c-HfNP nanoprobes in cell pellets; E) MD-applied SPCCT 3D rendering of the clot phantom; control and treated clots were placed in a 3D vasculature phantom for imaging; F) 3D SPCCT rendering of targeted clot showing delineated signals from binding of f-AuNPs (yellow) and calcium (white) showing clot calcification; G) 3D SPCCT rendering of control clot showing lack of AuNP signal; significant difference was visually observed in targeted c-HfNP and f-AuNP treatment groups vs nontargeted bare HfNP and AuNP treatment groups. The MARS MIQ algorithm was applied to accurately quantify nanoparticle binding across all treatment groups by analyzing K-edge signal pixels across 2D axial slices from the SPCCT scans. Graphs showing a significant increase in K-edge signal from H) targeted c-HfNPs in bEnd3 spheroid cells and (I) targeted f-AuNPs in the clot compared to the nontargeted and control groups showing binding specificity of both the nanoprobes. Data is presented as mean ± standard deviation (*N* = 3); **** p-value ≤ 0.0001.

Similarly, the targeted AuNPs were tested for their binding efficacy in a clot phantom model (Figure 5a). Since fibrin is not expressed in the cell line, we created an *ex vivo* clot phantom model using mouse blood plasma to mimic fibrous tissue in the hematoma membrane. Targeted f-AuNPs were treated with acellular fibrin clot phantoms and loaded into a 3D-printed vasculature phantom to mimic a thrombus expressing high levels of fibrin protein, which is a vital element of intravascular thromboses. These targeted clots were imaged using SPCCT along with control clots to visualize differential uptake of the targeted AuNPs. The control clots produced contrast similar to the surrounding vasculature with negligible Au signal (Figure 5g), whereas the targeted fibrin clot revealed a stronger attenuation obtained from the Au (yellow) and Ca (white) signals observed from the bound AuNPs and the calcification along the thrombus wall, respectively. The significant increase in the signal from bound f-AuNPs was analyzed (Figure 5e,f). Quantification of the dual nanoprobe binding in a μg amount of bound nanoparticles per treatment group was done using the MIQ algorithm across all 2D tissue slices, and voxel-wise maps of each nanoparticle material were generated in 3D (Figure 5h,i). The structural similarity between both the K-edge nanoparticles in terms of size, shape, and surface modifications allows similar binding efficacies to the target proteins of interest which is ideal for multiplexed molecular tissue imaging using SPCCT.

### Immunohistochemistry Evaluation of Patient Samples and ML Image Analysis and Quantification

Immunohistochemistry (IHC) analysis of multiple 2D tissue slices from each patient sample was initially conducted as an orthogonal validation for 3D tissue imaging using SPCCT (Figure 6a). The analysis provides a deeper understanding of the CSDH pathology of each patient sample and serves as a gold standard to verify the SPCCT MD data. While preparing 3D tissue blocks for CT imaging, a portion of the sample was fixed and sectioned to 5 μm-thin sections on glass slides. The tissues were immunostained for four distinct markers: vascular endothelial growth factor receptor 2 (VEGFR2), which facilitates the initiation of angiogenesis;^18,36^ platelet endothelial cell adhesion molecule-1 (PECAM), essential for microvasculature development;^37^ tyrosine kinase receptor (Tie2), expressed during the later stages of vascular development or quiescent adult vasculature;^38^ and fibrin, which is fundamental for clot stabilization,^39,40^ as illustrated in Figure 6b. Bright-field microscopy was used to assess the relative abundance of the molecular targets, and the resulting images were acquired generating a large data set for development of an ML algorithm for quantitative analysis (Figure 6c–e).

**Figure 6:**
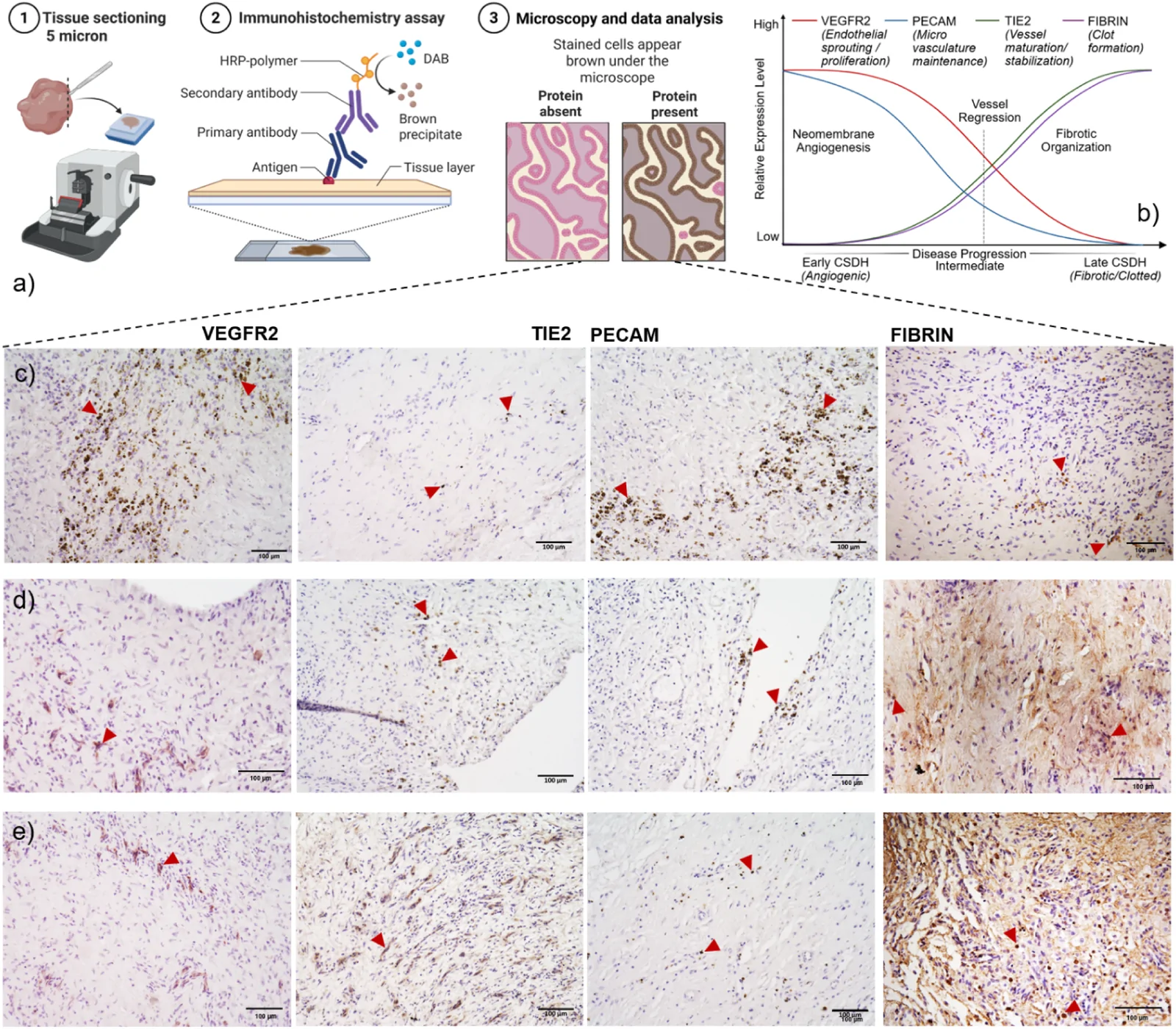
(a) Schematic showing immunohistochemistry analysis of patient tissue samples using a HRP–DAB sandwich assay followed by microscopy to visualize protein staining and expression profiles (created in BioRender); b) graphical representation showing expression trends of four relevant biomarkers for CSDH analysis: angiogenic factor VEGFR2 for early angiogenesis (new vessel growth), mature membrane factor TIE2 showing quiescence of angiogenesis, PECAM for microvasculature maintenance, and fibrin present in clotted fibrous tissues during different stages of disease progression (early angiogenesis to late fibrous stage) (created in BioRender). Bright-field microscopy images of CSDH tissue slices stained for the above four markers obtained from c) patient 1 showing high VEGFR2 and lower fibrin levels, scale bar = 100 μm. Slices also show cell nuclei due to H&E counterstaining; d) patient 2 showing comparable VEGFR2 and fibrin expression levels; e) patient 3 showing high fibrin expression and low VEGFR2 expression; comparative analysis of the expression profiles showed the patient 1 with an early angiogenic trend, the patient 2 with an intermediate trend, and a fibrous tissue composition for patient 3 with lesser angiogenic vessels and more mature membranes. Red arrows indicate the stained regions showing the presence of the target protein.

Although IHC is considered a gold-standard method for tissue analysis, it is largely qualitative and is not sufficient to validate SPCCT findings. Therefore, an in-house ML-enabled image analysis algorithm with a unique image-processing pipeline (Figures 7a and S4a) was developed to add quantification to the qualitative IHC data as a percentage area of immunostaining measured. A large data set was obtained by acquiring multiple nonoverlapping fields of view per tissue section during bright-field microscopy. The bright-field images were first preprocessed using color normalization to align all samples to a consistent color space and reduce interslide variability due to staining and illumination differences. The background was removed from each image, and the RGB image was transformed into an optical density space. Then, a two-stain color separation method was applied as a color deconvolution technique to separate the hematoxylin channel (cell nuclei) from the brown chromogen channel to generate a “brown-intensity” map. Brown chromogen signal regions were segmented using adaptive percentile thresholding (Otsu’s intensity-based thresholding) within the masked area. The resulting binary mask was refined using morphological opening/closing to reduce noise and improve connectivity. Overlay images were finally generated by superimposing the refined mask on the original image for visual verification. For each image block, the brown area percentage was computed as

$$Brown\;chromogen\;area\left(\%\right)=\frac{N_{mask}}{N_{region}}\times 100$$

**Figure 7:**
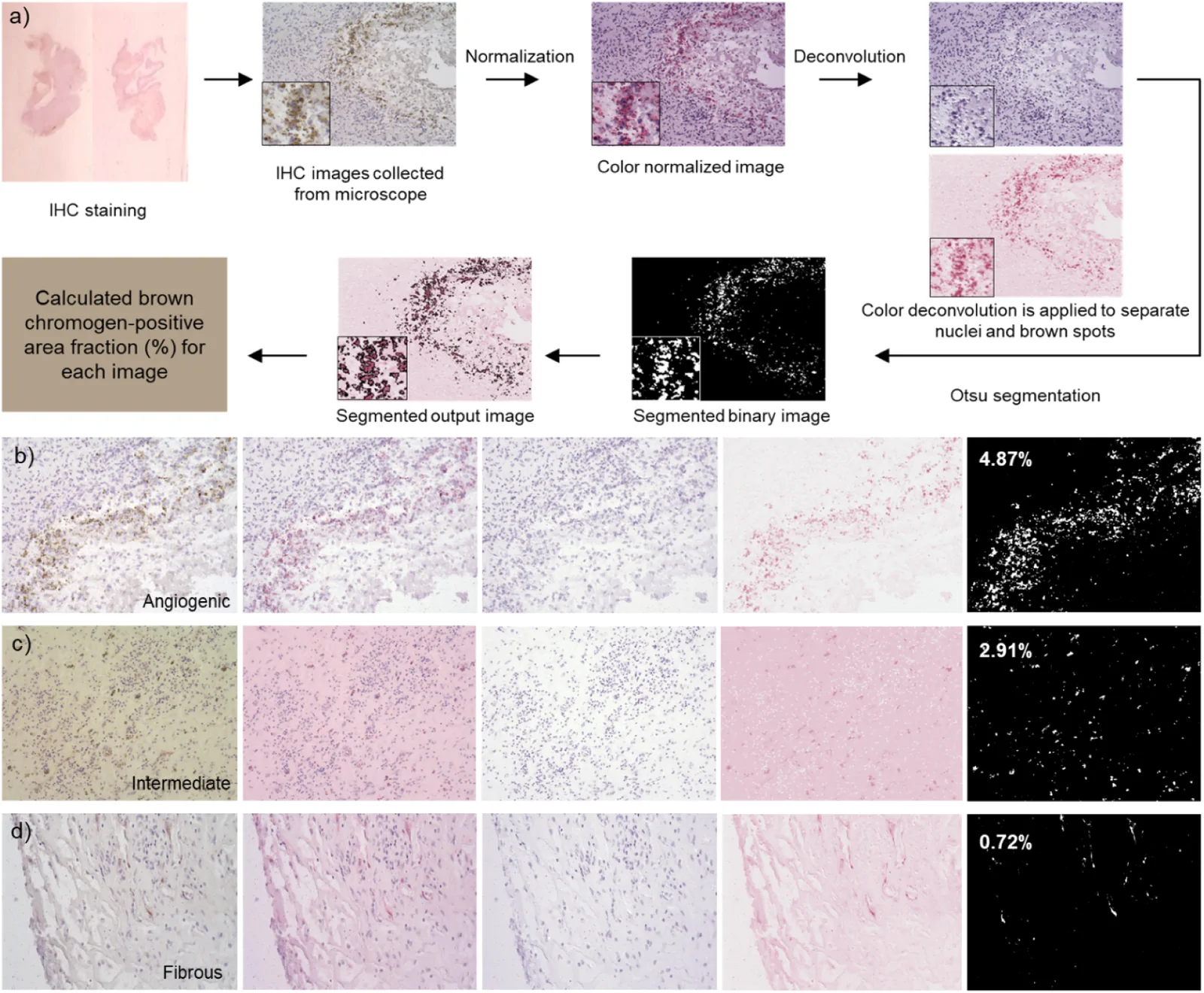
(a) Image processing pipeline used in the ML algorithm to segment brown signal regions from the IHC image data set and calculate the chromogen-positive % area fraction for quantification; IHC images showing segmentation and quantification of the VEGFR2-positive brown-stained regions across 3 different patient samples for comparative analysis: b) angiogenic sample from patient 1 showed the highest percent quantification of 4.87%, c) intermediate sample from patient 2 at 2.91%, and d) fibrous CSDH tissue with the lowest staining at 0.72% showing lower angiogenesis in the patient 3 sample.

where *N*_*mask*_ is the number of pixels labeled as brown in the binary mask and *N*_*region*_ is the total number of nonbackground pixels. When multiple blocks were available for one subject, the block-level percentages were averaged to obtain a single subject-level value, and the final result is reported as the mean across subjects. This quantification provided an objective, reproducible metric of IHC signal across all patient samples. Consistent with the SPCCT maps, IHC images (Figure 6c) and ML percent area quantification (Figure 7b) revealed regions of elevated angiogenic expression in the patient 1 sample that aligned with the higher HfNP (α_v_β_3_-targeted) signal (Figure 8b–e), while IHC/ML data from the later-stage patient 3 sample (Figures 6e and 7d) with more mature membrane expression that is characteristic of organized hematoma matched the SPCCT data showing increased AuNP (fibrin-targeted) signal (Figure 8j–m). This further demonstrates the accuracy of SPCCT-based material decomposition for simultaneous 3D mapping of α_v_β_3_- and fibrin-associated pathology in patient-derived hematoma tissues.

**Figure 8:**
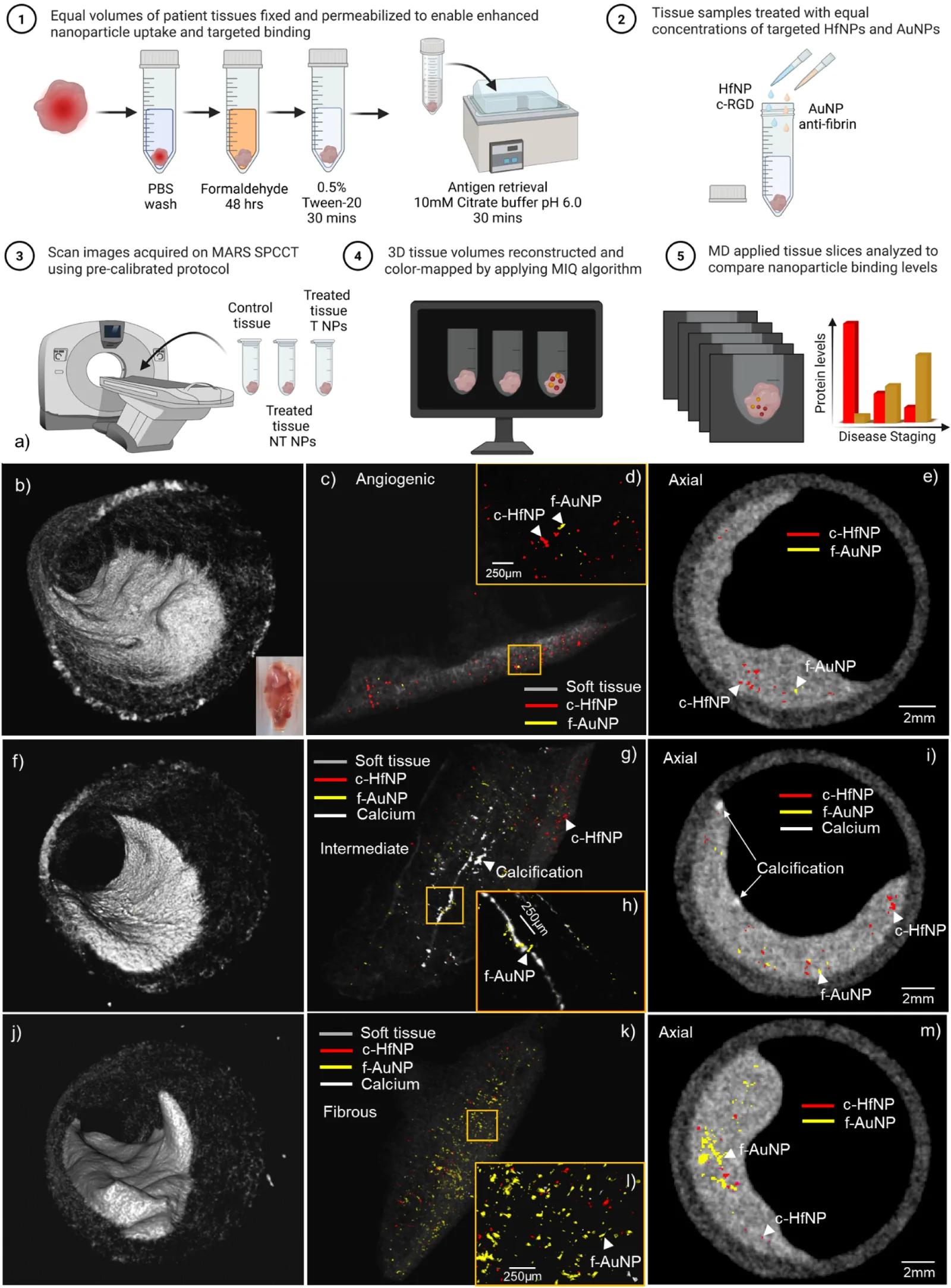
(a) Protocol for CSDH 3D tissue block processing, incubation with targeted nanoprobes, SPCCT imaging, and MD analysis for color mapping (created in BioRender); b) SPCCT 3D rendering of angiogenic CSDH tissue from patient 1 with the inset showing the preprocessed brain tissue. c) MD-applied SPCCT 3D rendering of the angiogenic CSDH tissue expressing high α_v_ß_3_ integrin expression levels visualized by the high c-HfNP signal (red). d) Magnified view of c-HfNPs and f-AuNPs signals in the tissue block following removal of the soft-tissue channel; scale bar = 250 μm. e) Axial SPCCT image showing a single slice of the angiogenic tissue with high HfNP binding (red) and comparatively lower AuNP signal (yellow); f) SPCCT 3D rendering of CSDH tissue from patient 2 exhibiting intermediate protein expression levels. g) MD-applied SPCCT 3D rendering demonstrating comparative α_v_β_3_ integrin and fibrin expression, visualized by the corresponding c-HfNP (red) and f-AuNP (yellow) binding signals. h) Magnified view of c-HfNPs and f-AuNPs binding signals; scale bar = 250 μm. Calcification (white) was identified by MD. (i) Axial SPCCT image showing individual material signals across the tissue; j) SPCCT 3D rendering of fibrous CSDH tissue from patient 3. k) MD-applied SPCCT 3D rendering demonstrating higher fibrin expression in clotted regions, visualized by high f-AuNP binding (yellow) and low c-HfNP binding (red). l) Magnified view of f-AuNP binding; scale bar = 250 μm. Axial SPCCT image showing high AuNP signal (yellow) and very minimal HfNP signal (red).

### SPCCT “Multicolor” Imaging and Quantification of 3D Tissue Blocks from Patient Samples

To enable multicontrast SPCCT assessment of dual nanoparticle uptake and targeting in freshly collected tissue samples, the hematoma tissue blocks were processed under standardized conditions and imaged as 3D volumes (Figure 8a). Equal volumes of tissue block from each patient sample were weighed out to enable cross-sample comparison which were immediately fixed and permeabilized in formaldehyde and 0.5% Tween-20 for enhanced nanoparticle penetration while preserving tissue integrity. Antigen retrieval for the entire 3D tissue block was performed using citrate buffer to enable protein activation for ligand binding prior to incubation with the targeted nanoparticles. The fixed tissues were incubated with equimolar concentrations of the targeted nanoparticles for 30 min and thoroughly washed with 1× PBS three times to remove unbound or loosely bound nanoparticles to reduce nonspecific signal. The incubation experiment was designed primarily to enable nanoparticle association, within α_v_β_3_-positive angiogenic vasculature, rather than endothelial internalization. This interpretation is consistent with the known biology of α_v_β_3_ integrin, which is enriched on activated angiogenic endothelial cells and can mediate ligand binding and, in some contexts, receptor-dependent uptake of cRGD-functionalized nanomaterials.^41,42^ Similar nanoparticle association is expected with fibrin-rich granulation tissue from f-AuNP incubation.^43^ Future studies using ultrastructural and cellular colocalization techniques are required to distinguish surface-bound from intracellular nanoparticle populations in the incubated tissues. The washed tissues were placed in Eppendorf tubes and imaged in an SPCCT scanner using the optimized protocol to visualize the targeted Hf and Au nanoparticle binding for ratiometric evaluation. MD was applied after reconstruction to extract multicolor 3D renderings of the tissue blocks in which c-HfNPs targeted to α_v_β_3_ integrin were displayed as a red signal, while f-AuNPs targeted to fibrin were displayed as a yellow signal as shown in Figure 8c,g,k. It was observed that tissue samples retrieved from patients at different stages of hematoma showed differential binding of c-HfNPs and f-AuNPs as expected due to the varying expression levels of the target proteins. Hematomas at the early angiogenic stage showed higher binding of c-HfNPs and low f-AuNP binding due to the high expression levels of α_v_β_3_ integrin (Figure 8c–e) while later-stage fibrous hematomas showed higher f-AuNP binding to fibrin that is overexpressed in clot formations (Figure 8k–m). Calcification or ossification was also noted in an intermediate patient sample with comparable particle binding, shown by the Ca (white) signal (Figure 8g–i), demonstrating that SPCCT tissue imaging can yield specific functional insights with comprehensive information on tissue composition. The multicolor MD maps were overlaid to get a visual confirmation of the distinct voxels occupied by the HfNPs and AuNPs to better understand their comparative distribution within the hematoma microenvironment. Control tissue blocks were scanned alongside targeted tissues in individual scans to threshold for background noise from the actual signal. The MIQ algorithm was applied to quantify metal content (in μg) across the whole 3D tissue block for individual patient samples, allowing comparison of the relative binding levels of both nanoprobes. Quantification was performed across multiple axial slices to capture all nanoparticle-containing voxels throughout the tissue block, enabling accurate assessment of their binding levels. The quantified Hf and Au probe signals were graphically represented as a ratio to assess the relative expression levels of their target proteins and evaluate tissue composition (Figure 9m). These SPCCT findings were further corroborated and matched with IHC imaging and ML quantification as shown in Figures S5, S6, and S7 for angiogenic, intermediate, and fibrous tissue compositions. The findings showed that SPCCT can be effectively used to track the expression trends of vascular activation/repair markers (α_v_β_3_) alongside clot composition (fibrin) markers and map them in the same patient-derived 3D tissue block to get molecular information about CSDH pathology and enable evaluation of targeted nanoparticle platforms in clinically relevant tissue samples.

**Figure 9:**
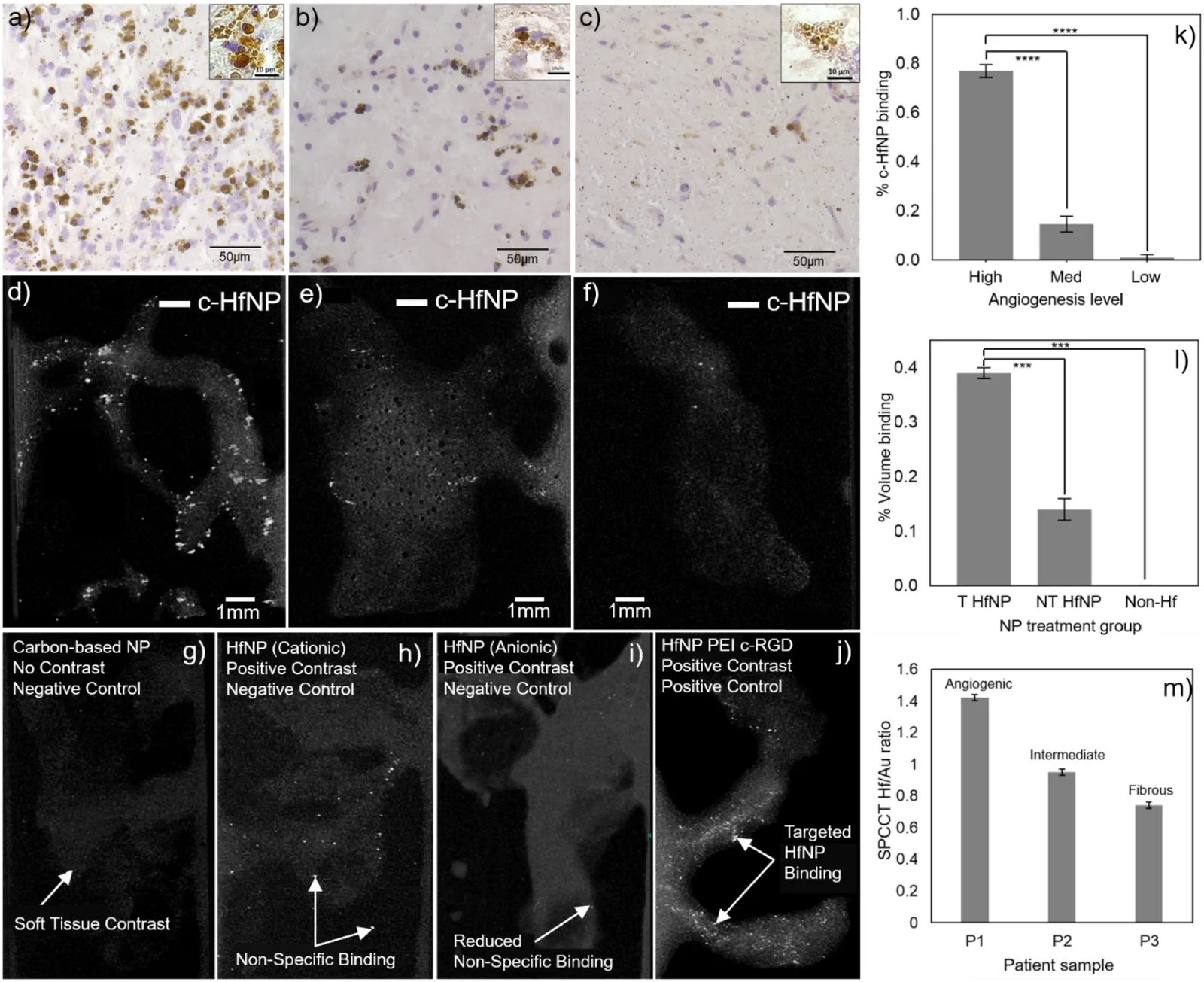
(a) Immunohistochemistry images of CSDH tissue slices showing a) high, b) moderate, and c) low VEGFR2 staining; scale bar = 50 μm; the insets show a 60× oil-immersion magnified image; scale bar = 10 μm; comparative microCT images of tissue sections from respective patient samples showing d) high, e) moderate, and f) low c-HfNP targeted binding (white signal) corroborated with the IHC data; scale bar = 1 mm; microCT data showing comparative analysis of CSDH tissue membranes treated with g) carbon-based nanoparticles as negative control, h) PEI-coated HfNPs, (i) succinic anhydride (SA)-coated HfNPs, and j) cRGD-coated HfNPs as positive contrast. The negative control showed no contrast from the carbon dots as expected. Nonspecific binding due to the positive surface charge of PEI-HfNPs is observed in the tissues which was reduced by the SA coating, and a significantly high signal was observed in tissues treated with cRGD-HfNPs showing successful targeted binding in the tissues; graphs showing k) percent c-HfNP binding from microCT data quantified across the three samples with significantly varying angiogenesis expression levels and l) percent volume binding of the nanoparticles quantified from microCT data in different treatment groups (carbon nanoparticles as negative control, nontargeted bare HfNPs, targeted c-HfNPs) showing binding specificity of the nanoprobes. c-HfNPs showed a significant increase in binding compared to other treatment groups; m) SPCCT-derived Hf/Au bound metal ratio in 3D whole-patient tissue samples with angiogenic, intermediate, and mature tissue composition imaged by a MARS scanner. Metal quantification was done using the MIQ algorithm post-material decomposition. Data is presented as mean ± standard deviation (*N* = 3); *** p-value ≤ 0.001, **** p-value ≤ 0.0001.

The architecture of CSDH tissue is considerably more heterogeneous than the *in vitro* model and may contribute to some degree of nonspecific nanoparticle retention due to steric barriers that affect nanoparticle transport and clearance. Therefore, a comparative study was conducted at the tissue level to support the *in vitro* findings using standard microCT, a widely used reference modality that provides high spatial resolution for 3D X-ray imaging of tissue specimens. The tissues were sectioned into 60 μm-thick slices and were treated with equimolar concentrations of both targeted and nontargeted nanoparticles as shown in Figures 9g–j and S8. While a low level of passive accumulation was observed, nontargeted nanoparticles consistently generated substantially weaker signals and less localized enrichment than their targeted counterparts. Quantitative analysis further demonstrated that targeted nanoparticles consistently exhibited preferential accumulation within target-associated regions, with a ∼3-fold increase in % bound signal in the tissues compared to the nontargeted particle signal (Figure 9l), supporting a significant contribution from specific ligand–target interactions beyond passive retention due to tissue heterogeneity. MicroCT tissue analysis was also done by treating samples with varying levels of angiogenesis with equimolar concentrations of c-HfNPs to observe differential binding. This data was corroborated with differential VEGFR2 IHC stain levels observed in the respective patient tissue samples as shown in Figures 9a–f and S9. Quantitative analysis showed a similar trend in CT signal levels with a significant increase in % binding in tissues expressing higher angiogenesis relative to samples with intermediate/lower expressions (Figure 9k). This further supports the SPCCT tissue analysis data.

## Conclusion

We demonstrate using human data that rationally engineered hafnium–gold K-edge nanopairs enable multiplex, voxel-level molecular mapping of intact CSDH surgical tissues using spectral photon-counting CT. By strategically positioning the nanoprobes within the diagnostic X-ray spectrum, we achieved minimized spectral crosstalk and accurate material decomposition for the simultaneous visualization of angiogenic and fibrotic microenvironments. The integration of surface-engineered high-Z nanomaterials with energy-resolved detector physics enables a quantitative framework for multicolor, volumetric phenotyping. This approach advances SPCCT beyond morphological imaging toward nanomaterial-enabled molecular tomography and provides a scalable platform for precision tissue characterization. By integrating spectral detector physics, rational nanomaterial engineering, and volumetric tissue analysis, this work enables a multiplexed K-edge imaging framework for quantitative molecular phenotyping of intact human surgical specimens. Beyond CSDH, this approach provides a generalizable strategy for nanomaterial-enabled multicontrast SPCCT across diverse pathologies characterized by spatially heterogeneous biomarker expressions.

## Materials and Methods

### Materials

All the chemicals and biological materials were purchased from reputable vendors and used without further purification steps. All solvents were of reagent grade and purchased from Sigma-Aldrich Inc. (St. Louis, MO, USA). Hafnium ethoxide (99.9%) was purchased from Alfa Aesar (Ward Hill, MA). Trimethoxysilylpropyl-modified (polyethylenimine) (1500–1800 g mol^–1^) was obtained from Gelest Inc. (Morrisville, PA). VEGFR2 monoclonal antibody (55B11) and CD31 (PECAM-1) monoclonal antibody (89C2) were purchased from Cell Signaling Technology, Inc. Tie-2/CD202b polyclonal antibody (19157–1-AP) was purchased from Proteintech. Antifibrin antibody (MAB2155) was purchased from Sigma-Aldrich. CycloRGD­(fk) (29514) was purchased from Cayman Chemical Company. All SignalStain reagents for immunohistochemistry studies were purchased from Cell Signaling Technology, Inc. For cell studies, a bEnd.3 frozen cell vial (CRL-2299) was purchased from ATCC and cultured in Gibco DMEM/FBS/Pen-Strep culture media obtained from Thermo Fisher Scientific. Thrombin (T6884) was purchased from Sigma-Aldrich in lyophilized powder form for *ex vivo* studies.

### Methods

#### Nanoprobe Synthesis and Surface Functionalization

Nontargeted hafnium oxide nanoparticles were synthesized in gram-scale quantities for this study using a water-in-oil microemulsion technique. Briefly, 300 mL of cyclohexane, 34.5 g of IGEPAL CO-520, 11.25 mL of ethyl alcohol, and 3.75 mL of NaOH (75 × 10^–3^ M) were stirred vigorously (500 rpm) under reflux conditions at 65 °C in a 500 mL round-bottom flask for 30 min to achieve homogeneity. 1.61 g of hafnium ethoxide powder was added to the above pot followed immediately with 750 μL of water, and the reaction progressed overnight during which HfO_2_ nanoparticles nucleated in the water-in-oil microemulsion. 7.5 g of trimethoxysilylpropyl-modified polyethylenimine was then added dropwise at a rate of 80 μL min^–1^ using a syringe pump and stirred continuously for another 2 h until the turbid suspension became transparent, indicating reaction completion. The solvent was evaporated in a rotary evaporator at 55 °C, and the polyethyleneimine silane-modified nanoparticles were washed with 100 mL of hexane:ethyl ether (50:50 vol/vol) once. The nanoparticles were washed with diethyl ether three times (50 mL each) and dried in a vacuum oven overnight. For ligand functionalization, cRGD­(fk) was conjugated via EDC-NHS chemistry onto succinic anhydride-modified HfNPs. A mixture of 3 mg mL^–1^ stock solution of modified HfNP with 0.06 mmol EDC, 0.05 mmol DMAP, and 2% cRGD­(fk) stock solution was stirred in a 20 mL scintillation glass vial for 48 h at room temperature. The mixture was centrifuged and washed to remove excess unbound antibody and resuspended in water and stored at 4 °C for further use in cell studies.

For AuNP synthesis, 100 mL of HAuCl_4_·3H_2_O (1.0 mM) was added to a beaker under stirring. The solution was heated to boiling, with continuous stirring. Then, 10 mL of trisodium citrate (38.8 mM) was rapidly added, and the mixture solution was boiled for an additional 20 min. As the mixture continued to boil, the heat source was turned off and maintained at room temperature. Sparkling ruby-red-colored AuNPs were produced and stored at 4 °C before use. For antibody functionalization of the AuNPs, 200 μL of DTSP solution (2.0 mM), a powerful cross-linking agent ideal for bioconjugation, was added to 100 mL of the prepared AuNP solution. The mixture was stirred for 45 min at room temperature. Antifibrin antibody was functionalized onto the Au-DTSP surface using EDC coupling by adding antibody in 1:200 dilution and stirring for 3 h at room temperature to prepare 30 mL of Au-DTSP-antifibrin NPs. Au-DTSP was bound to the amino group of fibrin antibody through its *N*-hydroxysuccinimide ester moiety, with the formation of an amide bond. The antibody-functionalized AuNP solution was centrifuged to remove excess unbound antibody from the supernatant. The purified particles were resuspended in DI water and stored at 4 °C before further use.

#### Dynamic Light Scattering

The average size distribution of HfNPs and AuNPs was measured by calculating their hydrodynamic diameter in water using a Malvern Nano-S Zetasizer. The particles were diluted to an optimum extent in disposable cuvettes before each measurement and multiplied by the dilution factor to obtain the results.

#### Transmission Electron Microscopy

5 μL of each nanoparticle suspension was drop-cast onto clean carbon-coated copper grids (400 mesh size). After 3 min, the excess liquid was wicked out using filter paper, and the grids were dried under vacuum overnight before imaging using the Hitachi HT7800 120 kV TEM with an AMT Nanosprint15 B digital camera at the UMBC campus. The anhydrous diameters of both HfNP and AuNP were calculated using ImageJ software.

#### Fluorescence Spectroscopy

Antibody conjugation to hafnium and gold nanoparticles was confirmed using FITC- and Cy5-based antibody labeling kits. Antibodies were fluorescently labeled according to the manufacturer’s instructions and subsequently conjugated to the nanoparticles using previously described surface coupling chemistry. Unbound antibodies and excess fluorophores were removed by repeated purification steps. Control nanoparticles processed with the labeling kits in the absence of antibody and untreated nanoparticles were prepared in parallel to assess nonspecific dye adsorption. Fluorescence emission spectra of antibody-conjugated and control nanoparticles were recorded in both solution and solid phases using a Horiba FluoroMax Plus fluorescence spectrophotometer under identical acquisition settings. The presence of characteristic FITC or Cy5 emission peaks exclusively in the antibody-conjugated nanoparticle samples confirmed successful antibody attachment and stable fluorophore association.

#### Inductively Coupled Plasma-Atomic Emission Spectroscopy (ICP-AES)

Hf and Au metal concentrations of the particles were measured using the Thermo Scientific iCAP 7400 spectrometer located at the Laboratory for Isotopes and Metals in Environments (LIME) facility at PSU. The samples were fully digested in aqua regia, and the final solutions were prepared as a 10x-diluted sample in 2% nitric acid to measure the metal concentrations.

#### Ligand Density Calculations

Nanoparticle number calculations were obtained using ICP-AES measurements. The total number of 10 nm-sized HfNPs was calculated to be ∼1.05544 × 10^12^ nanoparticles mL^–1^. The number of bound cRGD­(fk) molecules was calculated to be ∼3.31 × 10^12^ molecules mL^–1^ of HfNPs from the conjugation efficiency data. The number of bound ligands per nanoparticle was then obtained as,

$$\frac{\mathrm{Number of bound ligand molecules}/\mathrm{mL}}{\mathrm{Nanoparticle number}/\mathrm{mL}}$$

This yielded a ligand density of ∼3 cRGD­(fk) molecules per HfNP which corresponds to an estimated 2% surface coverage. Similarly, the total number of 10 nm-sized AuNPs was calculated to be ∼3.89957 × 10^14^ nanoparticles mL^–1^. The number of bound antifibrin molecules per AuNP was then optimized to achieve a nominal 1:1 nanoparticle-to-ligand ratio for targeted binding with minimal steric hindrance.

#### Human Tissue Samples

Chronic subdural hematoma membrane samples (*N* = 10) were collected from the University of Maryland Medical Center. The excised patient tissues were collected midsurgery directly from the operation rooms located at the Shock Trauma Center. Immediately upon excision, the tissues were handled using full personal protective equipment and transferred on ice to preserve the sample integrity. Specimens were stored at −80 °C and quickly processed until further analysis. All patient sample collections were performed following the protocol evaluated and approved by the Institutional Review Board (IRB) (approval number: HP-00096352) in accordance with ethical guidelines. Informed consent was waived.

#### Patient Study Design

This IRB-approved and HIPAA-compliant retrospective study was performed at the University of Maryland R. Adams Cowley Shock Trauma Center and included a consecutive cohort of patients admitted between January 2020 and June 2021. The inclusion criteria were (a) CSDH patients scheduled for membranectomy and (b) age 18 years. Patients were excluded if they presented with (a) concomitant parenchymal hematomas or extensive encephalomalacia and (b) recurrent CSDH with prior burr-hole evacuation or craniotomy.

#### Tissue Preparation for Imaging and Histology

3D tissue block permeabilization: Freshly collected hematoma tissue membranes were fixed in a 4% paraformaldehyde solution for 48 h and thoroughly washed with 1× PBS three times to remove any residual formaldehyde. Blotted tissues were weighed, and an equal mass (100 mg) from each patient sample was aliquoted for nanoparticle incubation and subsequent SPCCT imaging. These tissues were incubated in 1× PBS with 0.5% Triton X-100 for 30 min at room temperature to allow better penetration of buffers and ligands (antibodies, peptides). Antigen retrieval of these whole tissues was carried out since formaldehyde fixation creates cross-links that mask target antigens. Heat-induced antigen retrieval was carefully done to unmask the antigens while maintaining structural integrity. The tissues were submerged in a citrate buffer (10 mM, pH 6.0) and placed in a water bath at 70–80 °C for 30 min. Samples were heated uniformly without boiling to avoid tissue damage. The samples were then cooled to room temperature and incubated for an hour with 0.2% BSA in PBS to block any nonspecific binding. These tissue blocks were then incubated with nanoparticles in 50 mL centrifuge tubes under gentle shaking for 30 min. The samples were washed three times with 1× PBS to remove excess unbound nanoparticles to prevent nonspecific binding and imaged using SPCCT in 5 mL Eppendorf tubes.

2D tissue sections for antigen staining: Fixed tissue blocks from each patient sample were loaded into tissue cassettes placed in 70% ethanol for paraffin embedding. The paraffin-embedded samples were trimmed and sectioned using a microtome to obtain 5 μm-thick sections for immunohistochemical analysis and 60 μm-thick sections for micro-CT imaging. The 5 μm sections were mounted onto glass slides, while the thicker sections were collected and stored in 1 mL Eppendorf tubes for subsequent imaging. The thicker sections were processed further and incubated with the nanoparticles (targeted and nontargeted) in optical cuvettes and imaged using a Siemens Inveon small-animal microCT imaging system located at the Center for Translational Research in Imaging (CTRIM) facility at the University of Maryland School of Medicine.

#### Immunohistochemistry

5- μm thick tissue sections mounted on glass slides were deparaffinized using xylene and rehydrated through graded ethanol to water, followed by an antigen retrieval procedure to unmask the target antigens. Multiple tissue sections from each patient sample were stained for each of the target antigens, VEGFR2, PECAM, Tie2, and fibrin. Citrate antigen unmasking was done for all the targets except VEGFR2, by heating the slides in a microwave submerged in a 1× citrate unmasking solution until boiling. The slides were immediately removed and placed on a heat block at sub-boiling temperatures (95–98 °C) for 10 min. The slides were cooled to room temperature for 30 min. These slides were then incubated in 3% hydrogen peroxidase to quench endogenous peroxidase activity followed by 200 μL of 1× animal-free blocking solution to block nonspecific binding sites. Prior to incubation, the primary antibody solutions were prepared in the antibody diluent buffer at 1:600 (anti-VEGFR2), 1:2000 (anti-PECAM), 1:1000 (anti-Tie2), and 1:250 (antifibrin) against the target antigens. After washing, the sections were incubated with HRP-conjugated secondary antibodies. The brown stains were developed by addition of a chromogenic DAB substrate at the sites of antigen localization. Further counterstaining was done using hematoxylin to visualize the cell nuclei. The sections were dehydrated and coverslipped with a mounting medium for bright-field imaging. For VEGFR2, the EDTA antigen unmasking procedure was used where the slides were heated to sub-boiling temperatures (95–98 °C) for 15 min on a heat block and prepared for staining without cooling to room temperature. For staining, the ImmPACT NovaRED HRP substrate kit was used for antibody labeling at the sites of antigen localization. The processed slides were imaged using the Leica bright-field microscope with consistent acquisition settings (objective magnification (4×, 10×, 20×, and 60× oil-immersion), illumination intensity, exposure, and white balance) to enable reliable comparison between samples.

#### bEnd.3 Cell Culture and 3D Spheroid Model Generation

Mouse brain endothelial (bEnd.3) cells were cultured in a differentiation medium [Dulbecco’s Modified Eagle’s Medium with 10% FBS and 0.5% Pen-Strep] in a T-75 flask. The cells were incubated in a humidified atmosphere (37 °C, 5% CO2) until they reached confluency. The cells were passaged using 0.25% Trypsin-EDTA, and the medium was renewed every 2 to 3 days. Three-dimensional (3D) bEnd.3 were maintained in faCellitate Biofloat 96-well plates with 6000 cells/well. Spheroids were also cultured in DMEM containing 5% FBS, 1% sodium pyruvate, and 0.5% Pen-Strep. The spheroids were incubated with targeted nanoparticles for 2 h in a humidified environment. The cells were rinsed with 1× PBS to remove excess nanoparticles and pelleted in Eppendorf tubes for SPCCT imaging. Control 3D spheroids and spheroids treated with nontargeted HfNPs were also imaged to better visualize the targeted binding of c-HfNPs in targeted spheroids.

#### Ex Vivo Clot Phantom Model

Freshly collected whole mouse blood was centrifuged in Eppendorf tubes to separate the plasma. Clots were produced by adding 100 mmol/L calcium chloride (3:1 vol/vol) with 5 U of thrombin to the separated plasma. The plasma was allowed to coagulate slowly at room temperature. The plasma turns into a jelly-like substance showing successful formation of a strong fibrin mesh directly from fibrinogen due to a rapid clotting cascade. The clots were incubated individually with 150 μL of antifibrin-coated AuNPs in 10 mL of PBS for 2 h. Nontargeted clots were used as controls for SPCCT scanning. The clots were washed thoroughly with PBS to remove any excess unbound nanoparticles. Nontargeted clots were treated similarly with bare nanoparticles and used as controls for SPCCT imaging and thresholding. The washed clots were carefully placed in a 3D-printed vasculature phantom to mimic the actual clot environment for SPCCT imaging.

### Statistical Analysis

Statistical data were analyzed via GraphPad Prism 10.4.2. All plots were presented as mean ± standard deviation (SD) obtained after repeated measures. Statistical analysis was performed using a one-way repeated measure of analysis of variance (ANOVA) to evaluate assay response toward the various sample groups under investigation. Differences between experimental groups and control groups were considered statistically significant with a p-value <0.0001. The sample size applied for each experiment was indicated in the figure captions.

## Supplementary Material



## Data Availability

All data are available in the main text and the Supporting Information. The clinical samples were transferred as per material transfer agreements (MTAs). Additional data that supports the findings of this study can be provided upon reasonable request. Requests for additional resources should be submitted to dipanjan@psu.edu.

## Notes

Concentration-dependent fluorescence intensity curves and flow cytometry analysis for nanoparticle surface ligand conjugation quantification (Figures S1 and S2), SPCCT phantom linearity plots for calcium identification (Figure S3), ML image-processing pipeline for IHC chromogen quantification (Figure S4), ML vs SPCCT algorithm quantification corroboration based on patient tissue composition (Figures S5–S7), hematoma tissue sections tested for probe binding using microCT analysis (Figures S8 and S9), optimized SPCCT image acquisition parameters (Table S1), and SPCCT phantom % material quantification (Table S2) (PDF)
