# Supplementary material for: Multiplex K‑Edge Nanoprobe Imaging Enables 3D Volumetric Molecular Phenotyping of Human Hematoma Membranes
Source: ACS Nano. 2026 Sep 14;20(37):25318–34. doi: 10.1021/acsnano.6c05415 (PMC13602995; doi:10.1021/acsnano.6c05415)
Supplement: Supplementary file 1 [file nn6c05415_si_001.pdf]

Supporting Information

Multiplex K-Edge Nanoprobe Imaging Enables 3D Volumetric Molecular Phenotyping of Human Hematoma Membranes

Nivetha Gunaseelan[1,2,3], Pranay Saha[1,2], Moumita Moitra[3], Shraddha Krishnakumar[1,2], Parikshit Moitra[3], Uttam Bodanapally[4], Dheeraj Gandhi[5], Thorsten Fleiter[4], Bizhan Aarabi[6], and Dipanjan Pan [1,2,3]*

[1]Department of Biomedical Engineering, Nuclear Engineering, Materials Science and Engineering, The Pennsylvania State University, University Park, PA 16802, USA

[2] Huck Institutes of Life Sciences, The Pennsylvania State University, University Park, PA 16802, USA

[3]Department of Pediatrics, Centre of Blood Oxygen Transport & Hemostasis, University of Maryland Baltimore School of Medicine, Baltimore, MD 21201, USA

[4]Department of Diagnostic Radiology and Nuclear Medicine, University of Maryland Medical Center, Baltimore, MD 21201, USA

[5]Center for Metabolic Imaging and Therapeutics (CMIT), University of Maryland School of Medicine, Baltimore, MD 21201, USA

[6]Neurosurgery, University of Maryland Medical Center, Baltimore, MD 21201, USA

*Email of the corresponding author: dipanjan@psu.edu

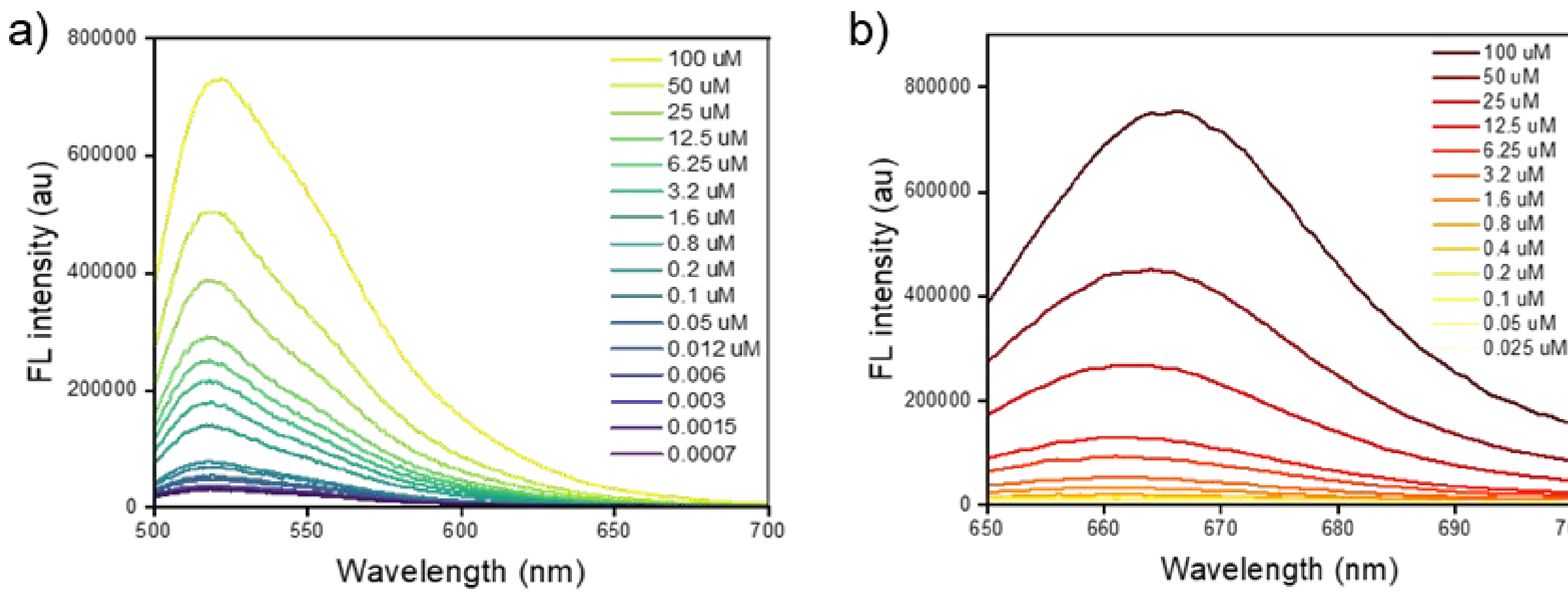

Figure S1 (a) Concentration dependent fluorescence intensity of FITC for ligand-conjugation determination with HfNPs (b) Concentration dependent fluorescence intensity of Cy5 for ligand-conjugation determination with AuNPs

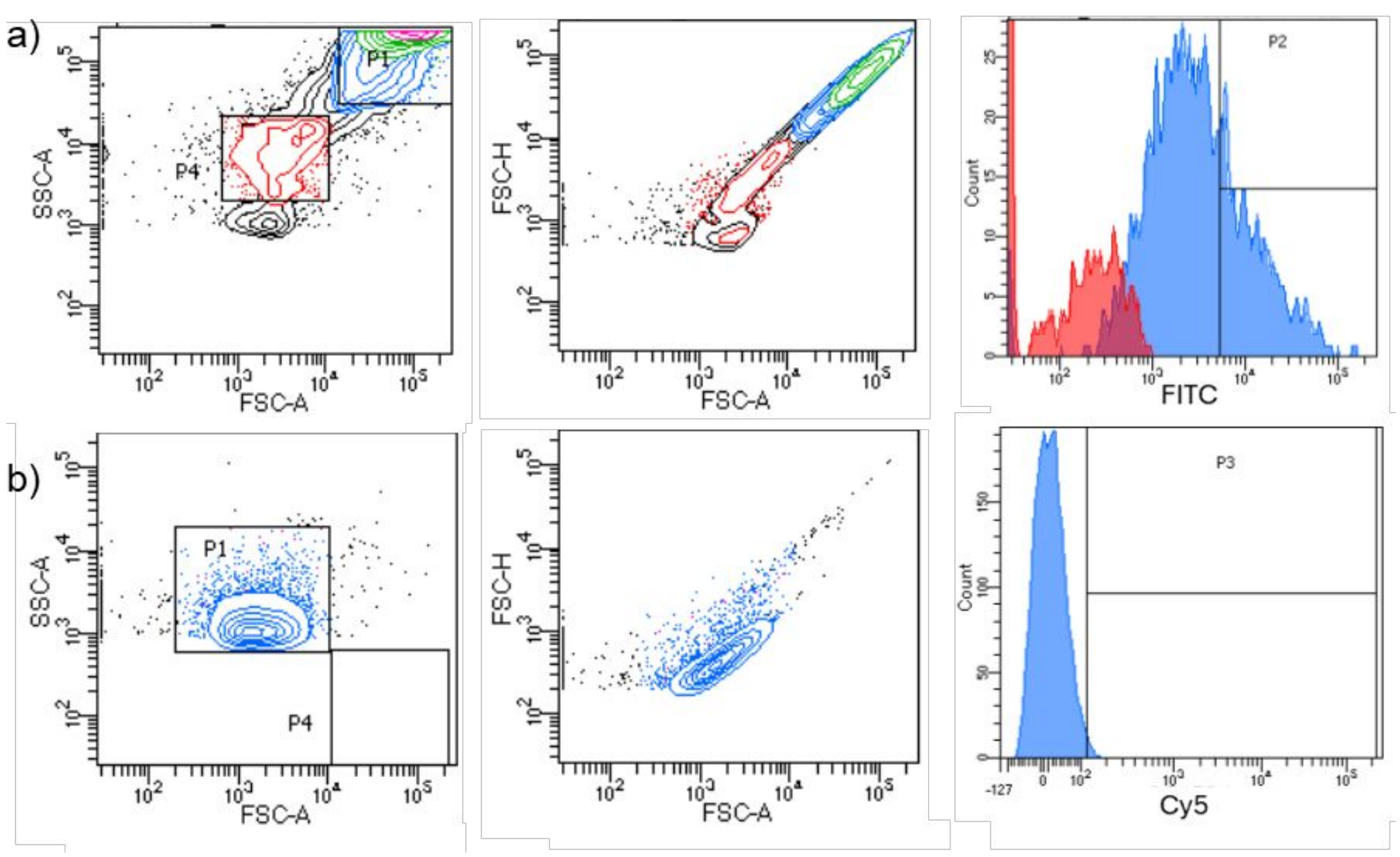

Figure S2 (a) 30% of FITC positive cells were found in unconjugated HfNPs compared to 68% of ligand-conjugated HfNPs, where the cellular autofluorescence or loosely adsorbed FITC to

HfNPs contribute to the signal in unconjugated HfNPs (b) Negligible % of Cy5 positive cells (%) were found for AuNPs without ligand conjugation

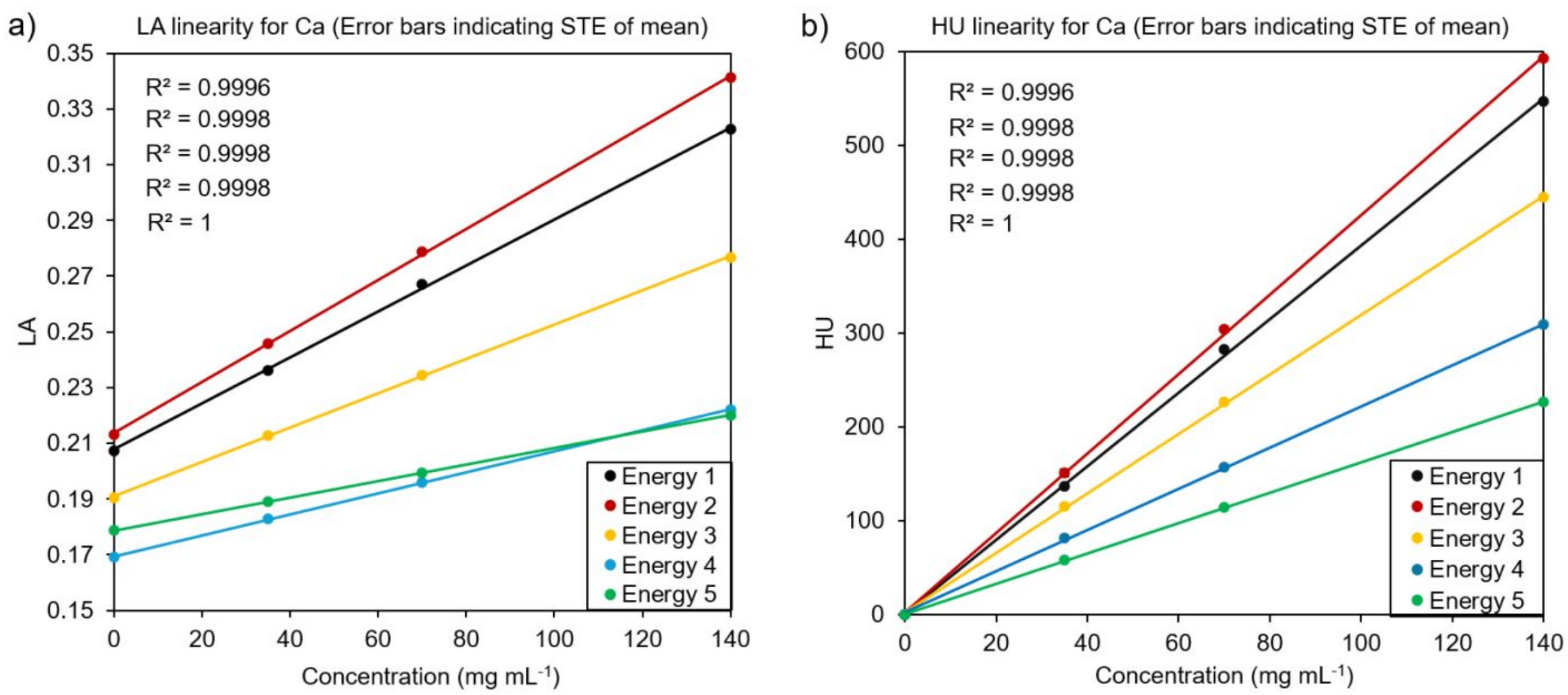

Figure S3 Phantom analysis plots showing linearity of attenuation of Ca in each energy bin f) Linear attenuation h) Hounsfield Unit across varying concentrations. The regression curves show that the SPCCT response is linear for the material demonstrating accuracy in identification. Data is presented as mean ± standard deviation (N=5)

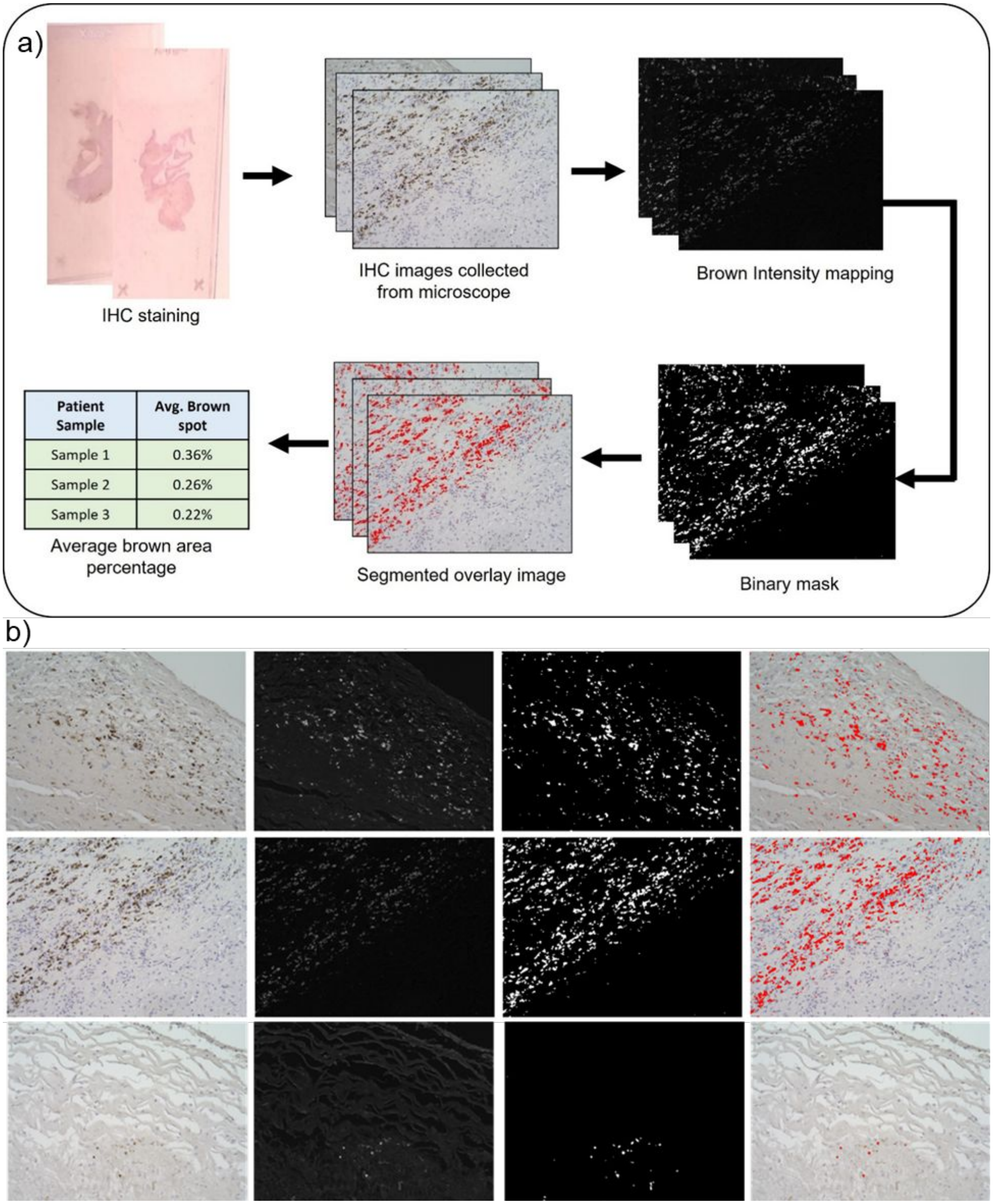
a)
IHC staining
IHC images collected
from microscope
Brown Intensity mapping
Binary mask
Segmented overlay image
Patient Sample
Avg. Brown spot
Sample 1
0.36%
Sample 2
0.26%
Sample 3
0.22%
Average brown area
percentage
b)

Figure S4 a) Workflow showing image-processing pipeline for chromogen brown-spot quantification in IHC images of CSDH membranes; b) Overlay images generated by superimposing the ML refined mask on the original IHC image for visual verification

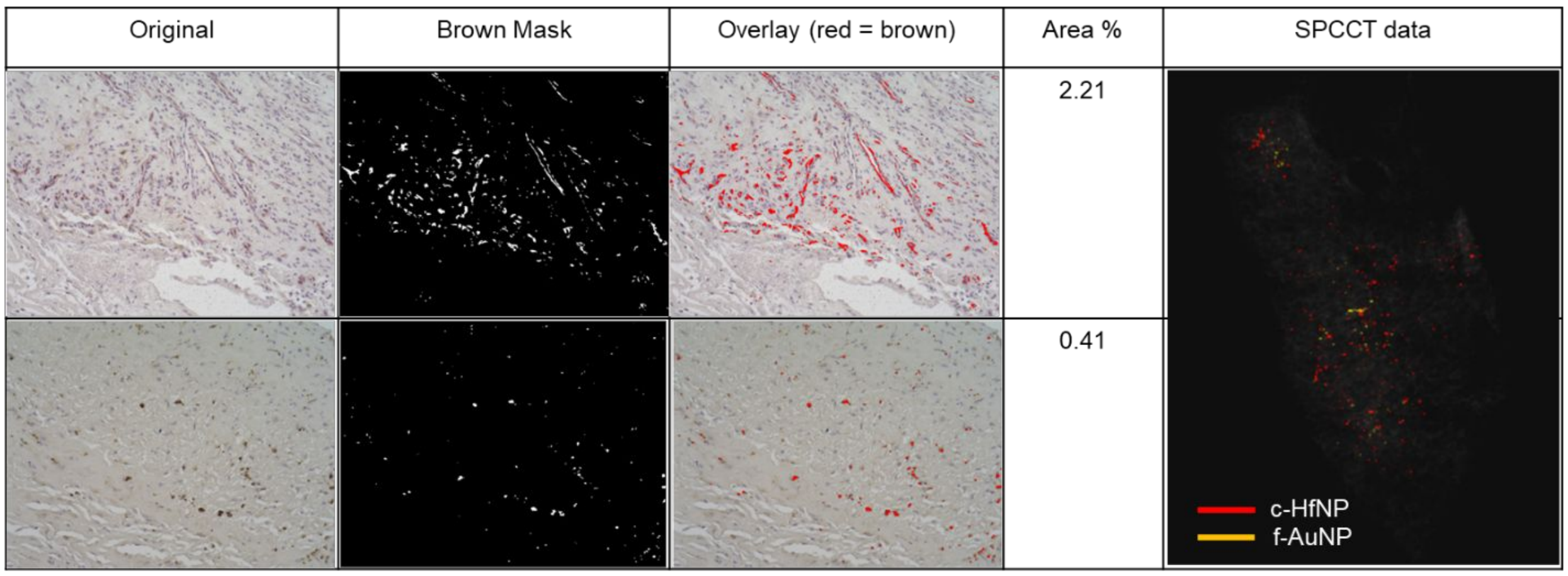

Figure S5 ML quantification (Area%) measured from IHC stained images corroborated with SPCCT data from a CSDH patient sample with angiogenic tissue composition. Images show high expression of HfNP bound to angiogenic factor and lesser AuNP bound to fibrin

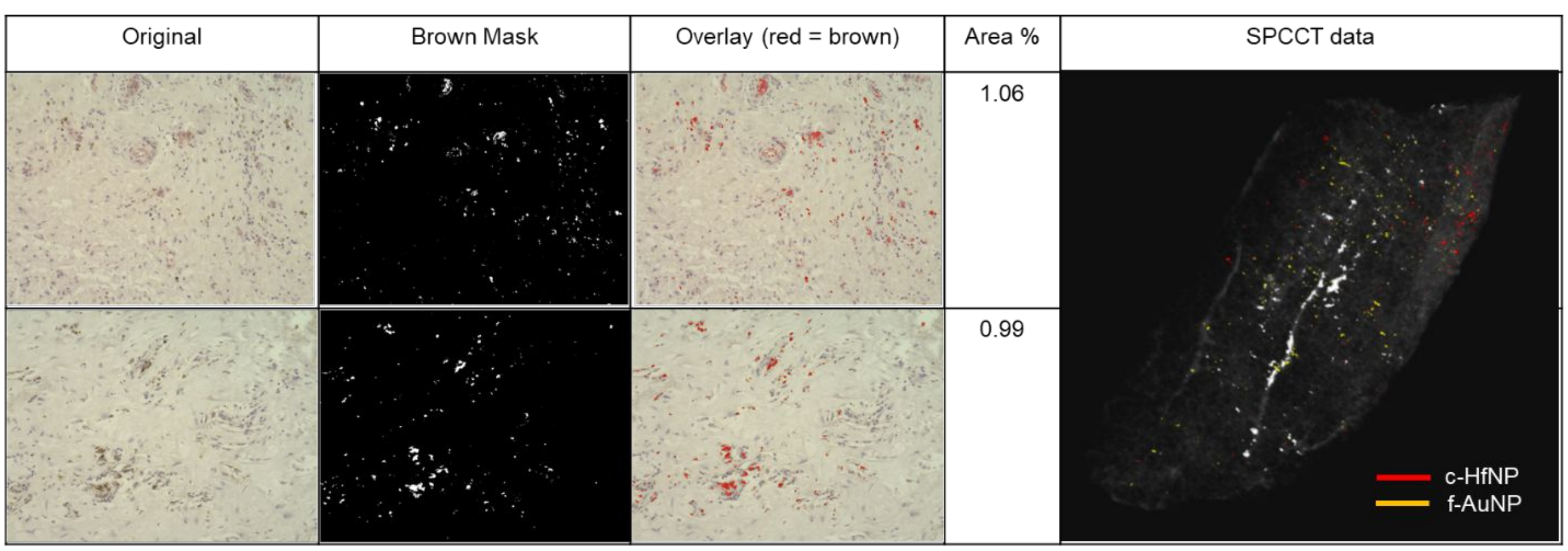

Figure S6 ML quantification (Area%) measured from IHC stained images corroborated with SPCCT data from a CSDH patient sample with intermediate tissue composition. Images show comparable expression of AuNP bound to fibrin and HfNP bound to angiogenic factor

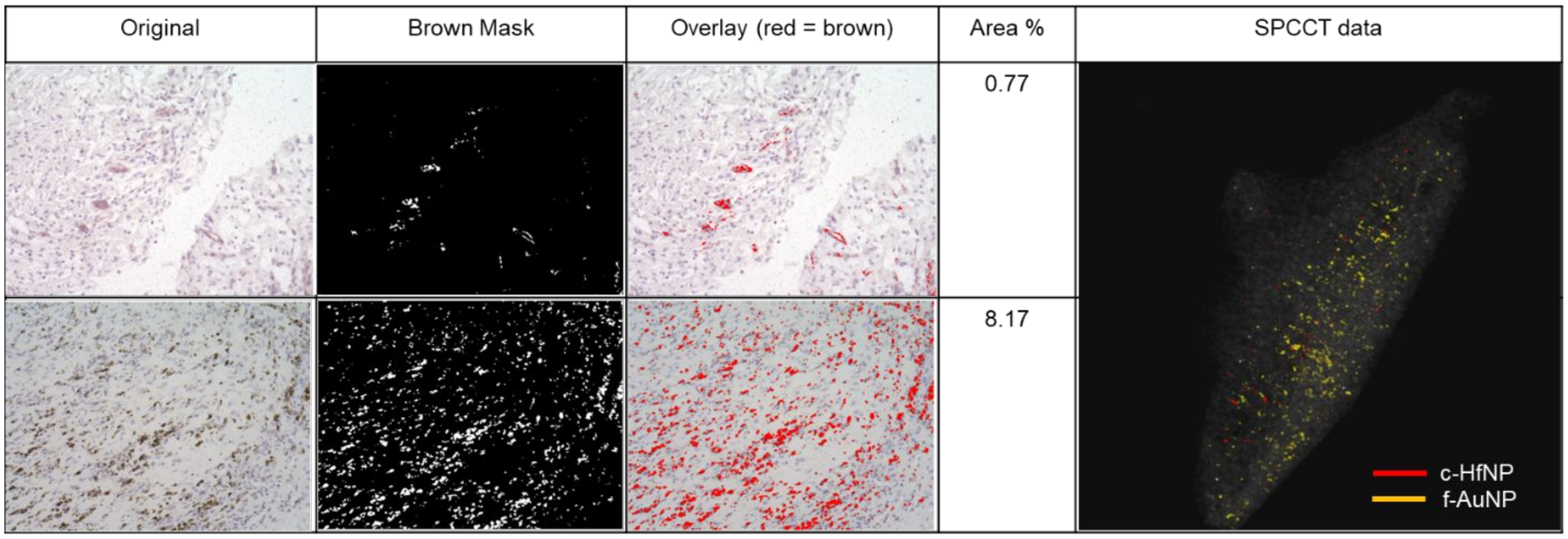

Figure S7 ML quantification (Area%) measured from IHC stained images corroborated with SPCCT data from a CSDH patient sample with fibrous tissue composition. Images show high expression of AuNP bound to fibrin and lesser HfNP bound to angiogenic factor

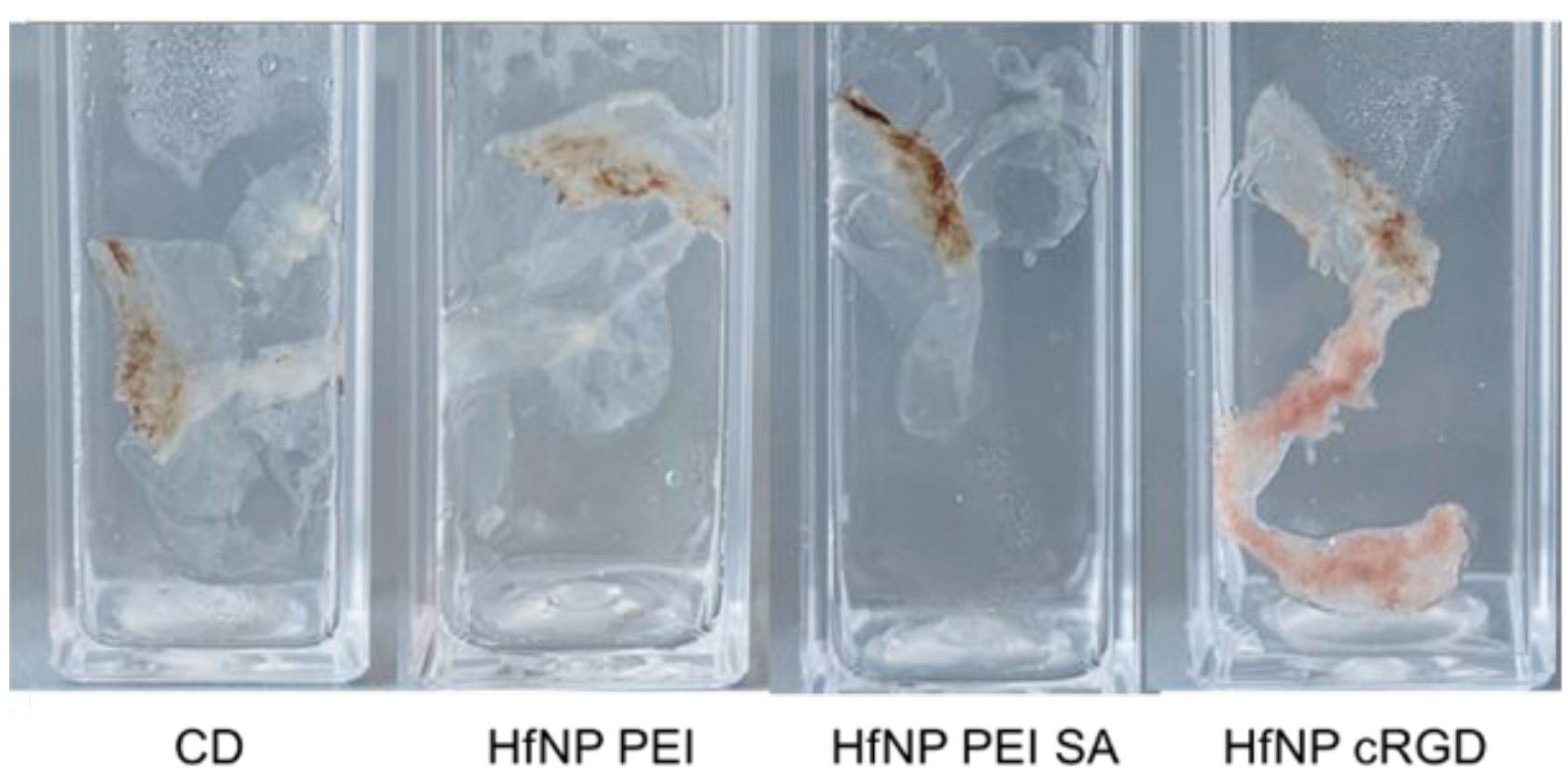

Figure S8 Images of cuvettes loaded with CSDH tissue membranes treated with carbon-based nanoparticles as negative control; PEI-coated HfNPs; succinic anhydride (SA) coated HfNPs; and cRGD coated HfNPs as positive contrast. These images correspond to the microCT data shown in Figure 9g-j.

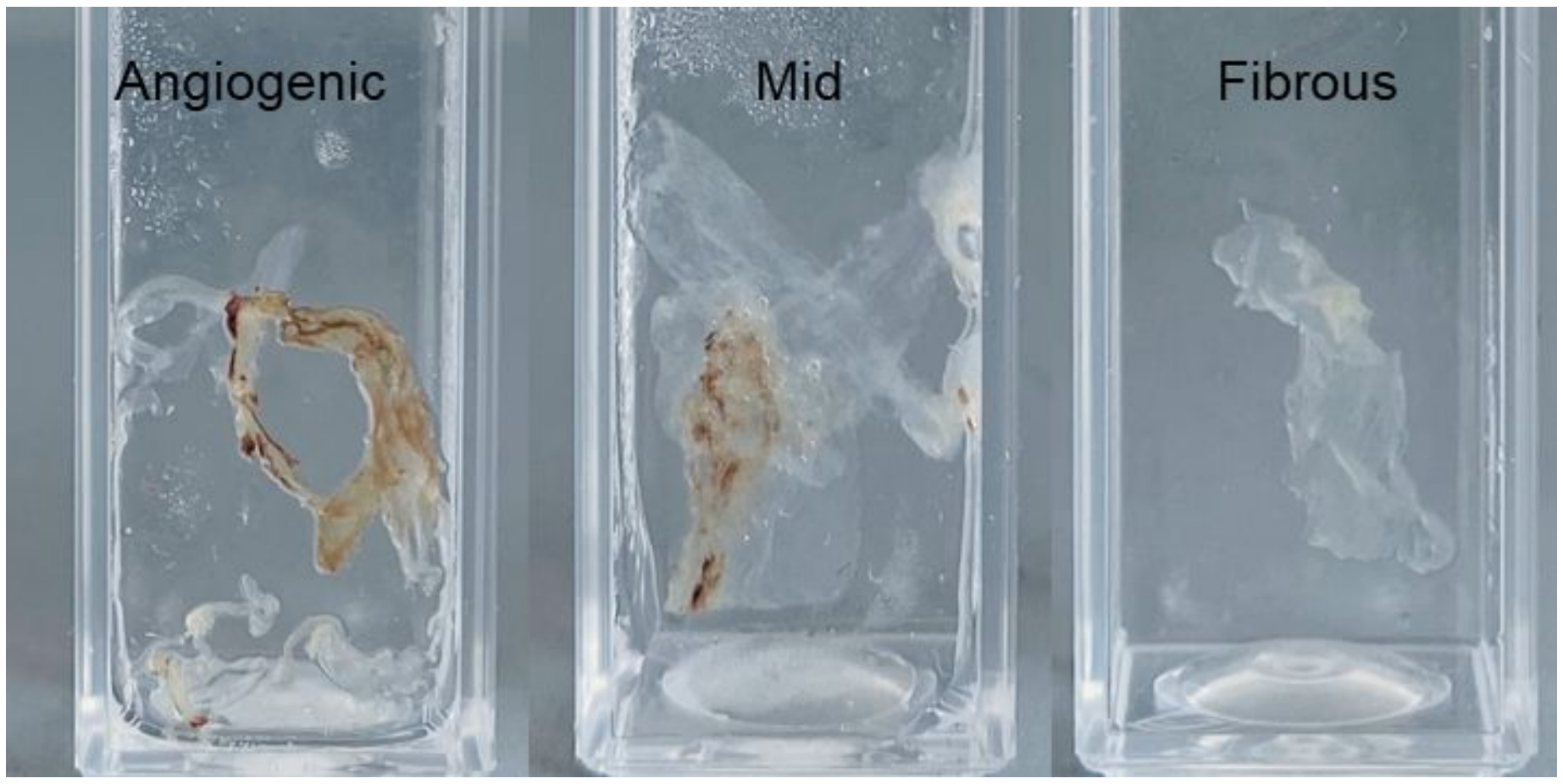

Figure S9 Images of cuvettes loaded with 60 μm thick CSDH membranes treated with cRGD-HfNPs and scanned using microCT

Acquisition parameters	Protocol Settings
Tube voltage	118 kVp
Tube current	61 μA
Exposure time	160 ms
Source detector distance (SDD)	274 mm
Source object distance (SOD)	199 mm
Circular projections, Flat fields	373 over 360°, 373
Voxel size	0.09x0.09x0.09 mm^3
Energy bins in Charge Sharing Mode (CSM)	7-35, 35-45, 45-55, 55-65, 65-118 keV

Table S1 Optimized SPCCT imaging protocol parameters to accurately detect both Hf and Au materials simultaneously

Material (mg mL^{-1})	Quantification (%)
Ca 35	86.9
Ca 70	92.1
Ca 140	98.0
Au 4.0	80.5
Au 8.0	80.7
Au 10.0	81.0
Hf 1.0	84.4
Hf 4.0	85.9
Hf 8.0	88.5

Table S2 Percent quantification of hafnium, calcium, and gold across various concentrations obtained from SPCCT phantom analysis using the optimized image protocol
